# Hypoxia-Initiated Supramolecular Free Radicals Induce Intracellular Polymerization for Precision Tumor Therapy

**DOI:** 10.1021/jacs.4c14847

**Published:** 2025-01-13

**Authors:** Mian Tang, Zhiqing Yang, Xingchen Tang, He Ma, Beibei Xie, Jiang-Fei Xu, Cheng Gao, David Bardelang, Ruibing Wang

**Affiliations:** † State Key Laboratory of Quality Research in Chinese Medicine, Institute of Chinese Medical Sciences, and MoE Frontiers Science Center for Precision Oncology, University of Macau, Taipa, Macau SAR 999078, China; ‡ Key Lab of Organic Optoelectronics and Molecular Engineering, Department of Chemistry, Tsinghua University, Beijing 100084, China; § School of Pharmacy, Shenzhen University Medical School, Shenzhen University, Shenzhen 518055, China; ∥ CNRS, ICR, AMUtech, Aix-Marseille University, Marseille F-13397, France

## Abstract

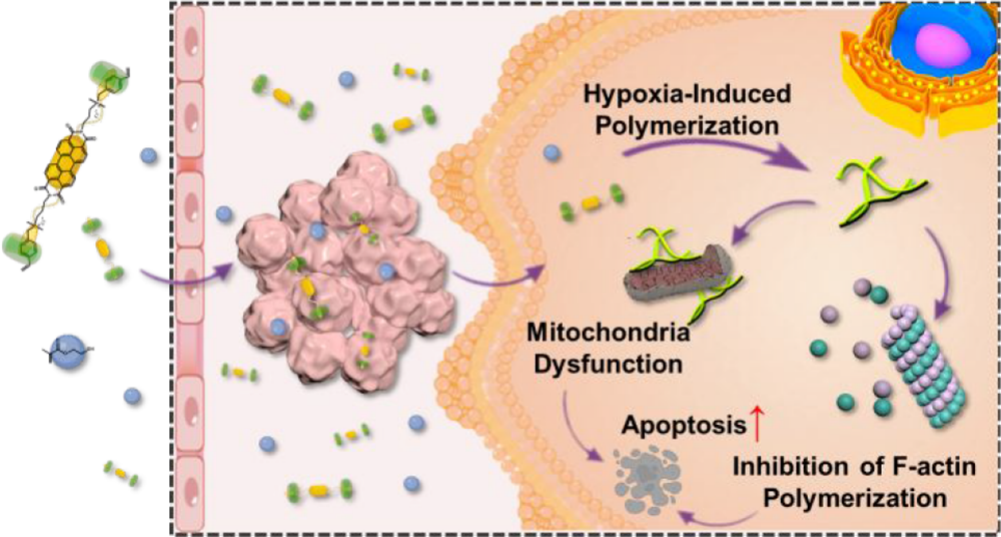

Despite the development of various controlled release systems for antitumor therapies, off-target side effects remain a persistent challenge. In situ therapeutic synthesis from biocompatible substances offers a safer and more precise alternative. This study presents a hypoxia-initiated supramolecular free radical system capable of inducing intracellular polymerization, thereby disrupting the cytoskeleton and organelles within 4T1 cells. The system utilizes a 2:1 supramolecular host–guest complex of cucurbit[7]uril (CB[7]) and perylene diimide derivative (PDI), termed PDI+2CB[7], which is selectively reduced by the tumor’s hypoxic and reducing environment to generate delocalized free radical anions. CB[7] effectively stabilizes these anions, enabling the PDI+2CB[7] complex to initiate free radical polymerization with 2-hydroxyethyl methacrylate (HEMA) inside the 4T1 cells. The resulting in situ polymerization significantly disrupts tumor metabolism, leading to a strong antitumor response without systemic toxicity. This study demonstrates that stable, endogenous stimulus-induced supramolecular free radicals can trigger intracellular polymerization reactions, achieving a selective and effective antitumor therapy without conventional chemotherapeutic agents.

## Introduction

Cancer remains one of the most lethal diseases globally, largely due to its challenging treatment, high metastatic potential, and frequent recurrence.^1^ Current antitumor strategies include chemotherapy, photodynamic therapy, sonodynamic therapy, radiotherapy, immunotherapy, and combination therapies.^2,3^ These methods, particularly when paired with advances in nanotechnology, aim to develop targeted and controlled drug release systems. While the unique size and properties of nanomaterials can improve drug delivery efficiency to specific targets, there remains a considerable risk of off-target effects during in vivo circulation.^4,5^ These off-target effects can lead to systemic toxicity, highlighting the urgent need for more precise tumor-targeting strategies.^6^

Utilizing biocompatible substrates for in situ synthesis of therapeutics within tumor cells offers a promising and precise antitumor strategy by directly influencing cancer cell life cycles, regulating cellular behaviors, and altering biological functions.^7^ For example, constructing polymeric materials within cells using biologically friendly substrates can effectively disrupt critical processes, such as the cell cycle, metabolism, cytoskeleton organization, intracellular material transport, and signal transduction, potentially leading to cancer cell death.^6,8−10^ This approach is considered both safe and efficient for tumor treatment.^11,12^

Polymerization occurs when carbon–carbon double bonds in molecular structures open under free radical attack, forming polymers.^13^ Most free radical polymerization reactions, however, require additional free radical initiators and often necessitate external triggers like UV irradiation^14,15^ or heating,^16,17^ which may inadvertently damage cells beyond the desired polymerization. These external stimuli make it difficult to study the direct impact of polymerization on cells. Moreover, the high reactivity of free radicals poses another challenge, as they tend to react with various cellular components, leading to a decreased activity and rapid quenching. Therefore, generating and stabilizing free radicals to minimize quenching while maintaining their activity within the tumor’s physiological environment remains a significant challenge.^18^

**Scheme 1:**
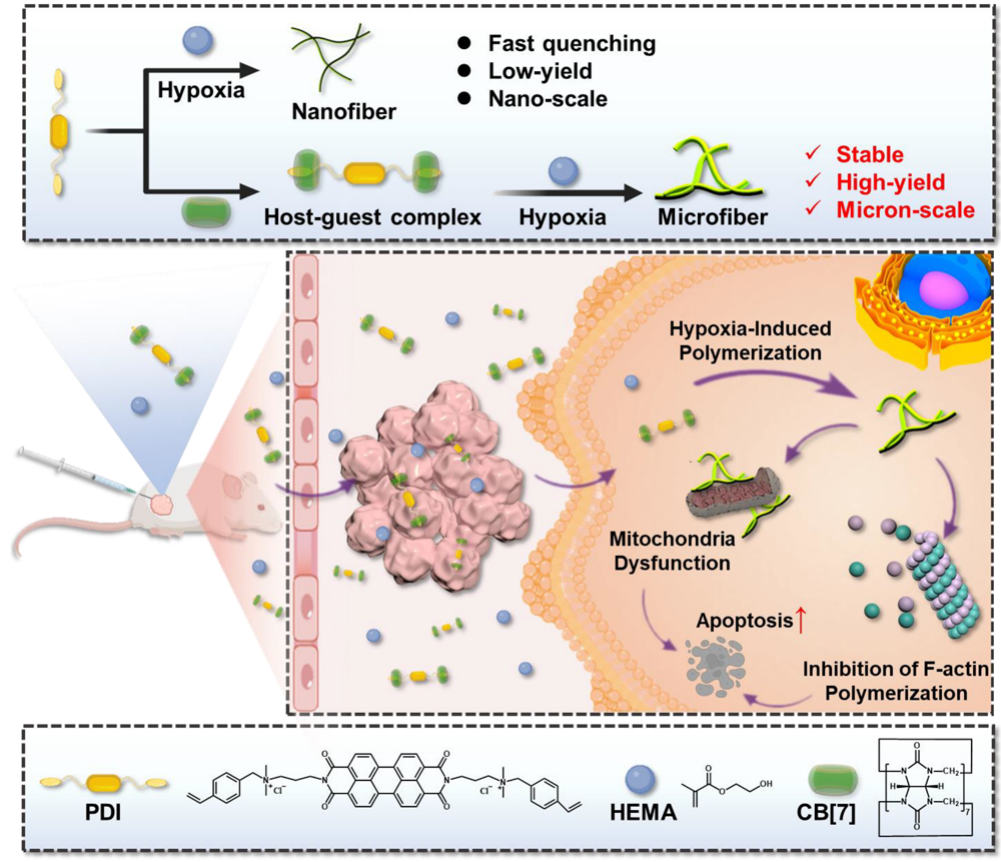
Schematic Illustration of Intracellular Polymerization Induced by Supramolecular Radical Anions in a Hypoxic Tumor Microenvironment

Tumors often thrive in oxygen-deficient environments,^19^ leading to the accumulation of reducing substances, such as glutathione (GSH),^20,21^ NAD(P)H,^22^ nitroreductase,^23^ and cysteine.^24^ In this study, we employed the supramolecular complex based on cucurbit[7]uril (CB[7]) and perylene diimide derivative (PDI), PDI+2CB[7], to generate delocalized free radical anions in response to the tumor’s reducing environment. CB[7], through its host–guest complexation with PDI, stabilized the supramolecular free radicals, enhancing their intracellular production yield.^25^ This triggered polymerization reactions with the biocompatible monomer 2-hydroxyethyl methacrylate (HEMA), leading to the in situ formation of polymers within tumor cells. The resulting polymerization disrupted the cytoskeletal actin and impaired organelle function while demonstrating minimal toxicity to noncancerous cells. In vivo investigations confirmed intracellular polymerization and long-term retention of these polymers in tumor tissues, exhibiting significant targeted antitumor efficacy (Scheme 1). This hypoxia-initiated supramolecular free radical-induced polymerization presents a promising, nonchemotherapy approach for precise antitumor therapy.

## Results and Discussion

**Figure 1:**
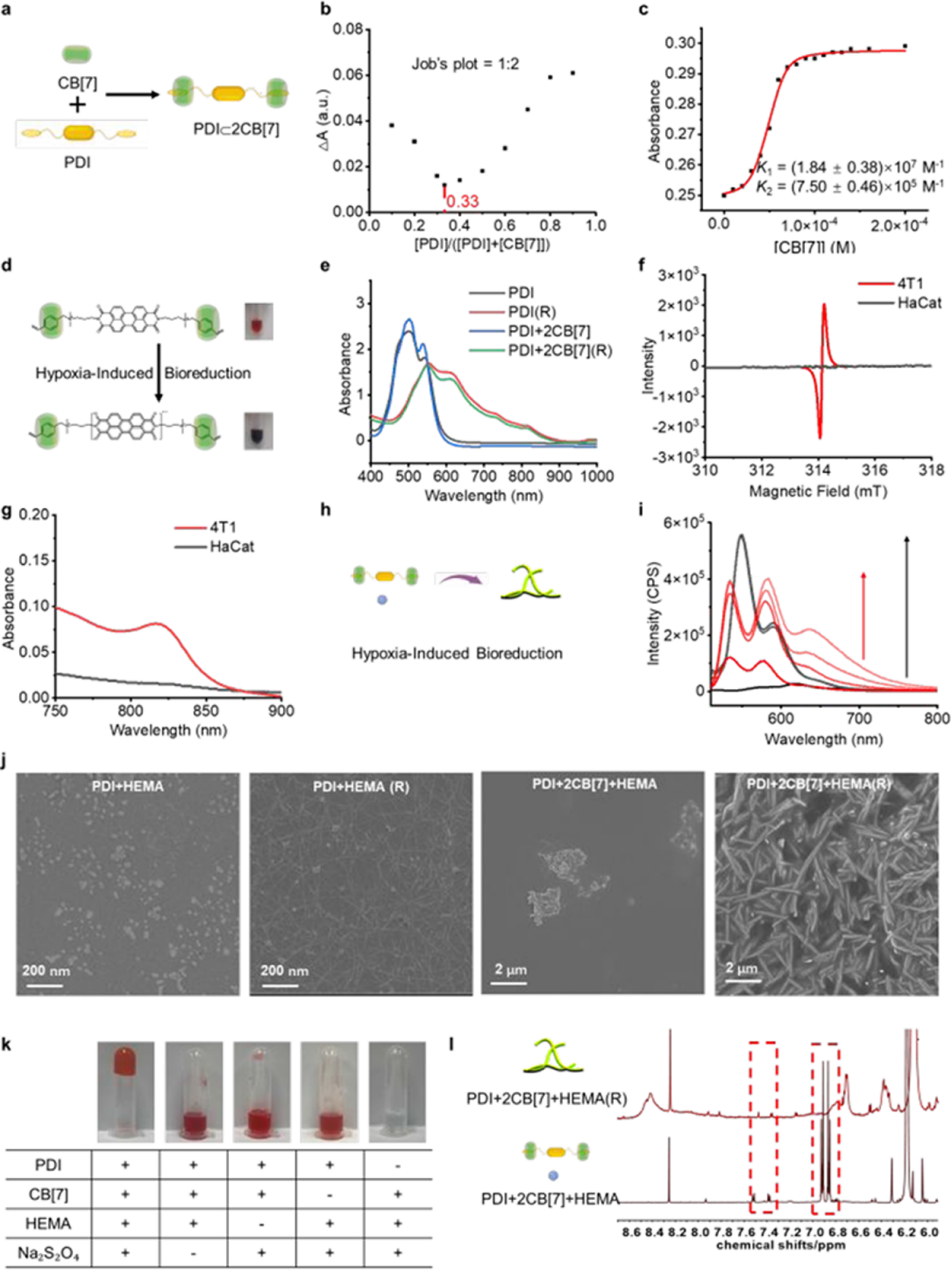
Formation of host–guest complexes and supramolecular radical-initiated polymerization in a reducing environment. (a) Schematic diagram of the assembly process of PDI and CB[7]. (b) Job’s plot of PDI+2CB[7] complexation ([PDI]+[CB[7]] = 5.0 μM). (c) Nonlinear least-squares analysis of the differential UV–vis absorbances (Δ*A*) to calculate the *K*_S_ values. (d) Schematic diagram of the process of generating free radical anions in a reducing environment of hypoxia. (e) Full UV–vis spectra of PDI, PDI+2CB[7], PDI radical anions, and PDI+2CB[7] radical anions in aqueous solution. [CB[7]] = 2[PDI] = 1.0 mM and [Na_2_S_2_O_4_] = 2.0 mM. R: reducing agent, Na_2_S_2_O_4_. (f) EPR spectra of the supramolecular PDI radical anions generated from PDI+2CB[7] complexation in the presence of 4T1 cells under hypoxic conditions and HaCat cells under normoxic conditions. [CB[7]] = 2[PDI] = 10.0 μM. (g) UV–vis spectra of supramolecular PDI radical anions in the presence of normoxic HaCat cells and hypoxic 4T1 cells after 12 h of culture. [CB[7]] = 2[PDI] = 10.0 μM. (h) Schematic diagram of the polymerization process in a reducing environment of hypoxia. (i) Fluorescence emission spectra of PDI+HEMA (black line) and PDI+2CB[7]+HEMA (red line) in a reducing environment of hypoxia in 1 h. [CB[7]] = 2[PDI] = 2[HEMA] = 1.0 mM, [Na_2_S_2_O_4_] = 2.0 mM (R: reducing agent, Na_2_S_2_O_4_). (j) Scanning electron microscopy (SEM) images of PDI+HEMA and PDI+2CB[7]+HEMA in a normal environment or in a hypoxic, reducing environment. [CB[7]] = 2[PDI] = 2[HEMA] = 10.0 μM, [Na_2_S_2_O_4_] = 20.0 μM. (k) Optical images of samples in a normal environment or in a reducing environment of hypoxia. [CB[7]] = 2[PDI] = 2[HEMA] = 1.0 mM, [Na_2_S_2_O_4_] = 2.0 mM. (l) ^1^H NMR spectrum (600 MHz, 90% D_2_O, 10% DMSO-*d*_6_, 25 °C) of PDI+2CB[7]+HEMA and PDI+2CB[7]+HEMA(R) (R: added Na_2_S_2_O_4_ as a reducing agent).

To prepare a free radical generator in a biologically reducing environment, PDI was first synthesized through a sequential aldimine condensation reaction between 3,4,9,10-perylenetetracarboxylic dianhydride and *N*,*N*-dimethyl-1,2-ethanediamine and a halogenation reaction between the resulting 2,9-bis(3-(dimethylamino)propyl)anthra[2,1,9-*def*:6,5,10-*d*′*e*′*f*′]diisoquinoline-1,3,8,10(2*H*,9*H*)-tetraone and 4-vinylbenzyl chloride. Subsequently, the three doublet peaks between 5.3–6.7 ppm demonstrated the successful modification of the terminal double bonds in the PDI structure, as shown in ^1^H NMR spectroscopy and further illustrated by the results of ^13^C NMR spectroscopy and high-resolution mass spectrometry (HRMS) (Figures S1–S4). Due to the existing benzene ring group and adjacent ammonium, CB[7] binds with PDI, and the host–guest binding behaviors in an aqueous solution were investigated by ^1^H NMR spectroscopy. As discerned from Figure S5, with the addition of 2 equiv of CB[7], the benzyl proton resonances of PDI shifted significantly to a high field, indicating that the benzene ring in the PDI structure was exclusively included in the cavity of CB[7]. Simultaneously, the proton resonances on the vinyl group of PDI (around 6.0 ppm) shifted downfield along with the weakening of π–π stacking. To elucidate the proton changes clearly, the correlation spectroscopy (COSY) of PDI and PDI+2CB[7] was analyzed (Figures S6 and S7) to determine the chemical shifts corresponding to the protons in the structure. Job’s plot further validated the 1:2 complexation stoichiometry between PDI and CB[7] via UV–vis spectroscopic titration in Figure 1b, consistent with the inference in Figure 1a. The stepwise binding constants (*K*_1_ and *K*_2_) were calculated to be 1.84 × 10^7^ and 7.50 × 10^6^ M^–1^, respectively, by the nonlinear least-squares curve-fitting method (Figures 1c and S8), suggesting a strong binding affinity.

To verify the generation of free radical anions under a reducing environment, sodium dithionite (Na_2_S_2_O_4_) (in borate buffer, pH = 8) was chosen to simulate the in vitro reducing environment.^26,27^ When freshly prepared Na_2_S_2_O_4_ solution was added into an EP tube with a lid, the PDI+2CB[7] solution underwent a distinguished color change from red to blue after stirring for 3 min (Figure 1d), indicating the potential formation of PDI free radicals. Moreover, UV–vis spectroscopy demonstrated that the intensity of characteristic absorption peaks of both PDI and PDI+2CB[7] at 495 and 543 nm decreased after the addition of Na_2_S_2_O_4_, accompanied by the increase in absorbances at 734 and 818 nm, respectively (Figure 1e). These results suggested that both PDI and PDI+2CB[7] solutions could generate free radical anions in response to the reducing environment. Furthermore, the bioreduction process of PDI+2CB[7] was analyzed by electron paramagnetic resonance (EPR) spectroscopy. As shown in Figure 1f, the EPR signal with a *g* factor of 2.0045 could be clearly observed in the presence of tumor cells (mouse breast cancer cells, 4T1 cells) under hypoxic conditions, while there was no typical EPR signal in the presence of noncancerous cells (human immortalized epidermal cells, HaCat cells) under normoxic conditions, confirming the formation of supramolecular radical anions in response to bioreduction environment. Also, 4T1 cells cultured in a hypoxic environment showed a characteristic absorption band of supramolecular free radical anions at 818 nm after treatment with PDI+2CB[7] (Figure 1g). In contrast, the UV–vis absorption signal of free radical anions could not be observed in HaCat cells under normoxic conditions. These results exhibited that supramolecular free radical anions could only be triggered from PDI+2CB[7] in hypoxic 4T1 cells rather than normoxic HaCat cells.

Due to the cross-linked free radical polymerization reaction, HEMA with carbon–carbon double bonds displayed the potential for polymerization in the presence of the PDI+2CB[7] complex under a hypoxic environment (Figure 1h). With the addition of Na_2_S_2_O_4_, the fluorescence of PDI+HEMA disappeared completely due to the formation of free radical anions under reducing conditions (Figures 1i and S9). With the extension of time, a weak fluorescence was observed, which could be attributed to the occurrence of polymerization reactions caused by free radicals. Of note, when CB[7] was added to the system, the fluorescence intensity of PDI+HEMA was significantly enhanced by 1.7 times, resulting from the fact that the supramolecular complexation weakened the π–π stacking of free PDI. Thus, the speed and extent of free radical production and polymerization reaction were significantly improved. It was consistent with previous report that CB[7] could weaken the close stacking of perylene diimide^28^ and suppress the dimerization and quenching of perylene diimide radical anions, ultimately improving the free radical yield in an aqueous solution. Meanwhile, scanning electron microscopy (SEM) imaging showed that PDI+2CB[7]+HEMA formed obvious microscale fiber polymers from blocky substances in a reducing environment (Figure 1j), while PDI+HEMA could only form nanoscale fiber polymers, further indicating the importance of CB[7] to promote free radical polymerization driven by a reducing environment. When the concentration of PDI+2CB[7]+HEMA was increased to the millimolar level, the formation of hydrogel was even observed after incubation for 4 h, whereas the mixture of PDI+2CB[7], HEMA, or Na_2_S_2_O_4_ could not induce the gelation process (Figure 1k). In addition, ^1^H NMR spectroscopy of PDI+2CB[7]+HEMA and PDI+HEMA showed that the proton of carbon–carbon double bonds at the end of the PDI structure was significantly reduced in a reducing environment (Figures 1l and S10), confirming that these unsaturated bonds were opened under the attack of free radicals and participated in the polymerization with HEMA. At the same time, the integration of carbon–carbon double bonds at 6.8–7.0 ppm in ^1^H NMR spectroscopy of PDI+HEMA(R) was moderately reduced; in the presence of CB[7], the peak of carbon–carbon double bonds almost disappeared, and a new peak was obviously generated at 6.6–6.9 ppm, indicating that the polymerization efficiency was significantly increased in the presence of CB[7]. Consequently, this evidence sufficiently proved that PDI+2CB[7] could be reduced to generate stable free radical anions in a hypoxic environment, thereby opening the carbon–carbon double bonds to polymerize with HEMA and finally generate polymers.

**Figure 2:**
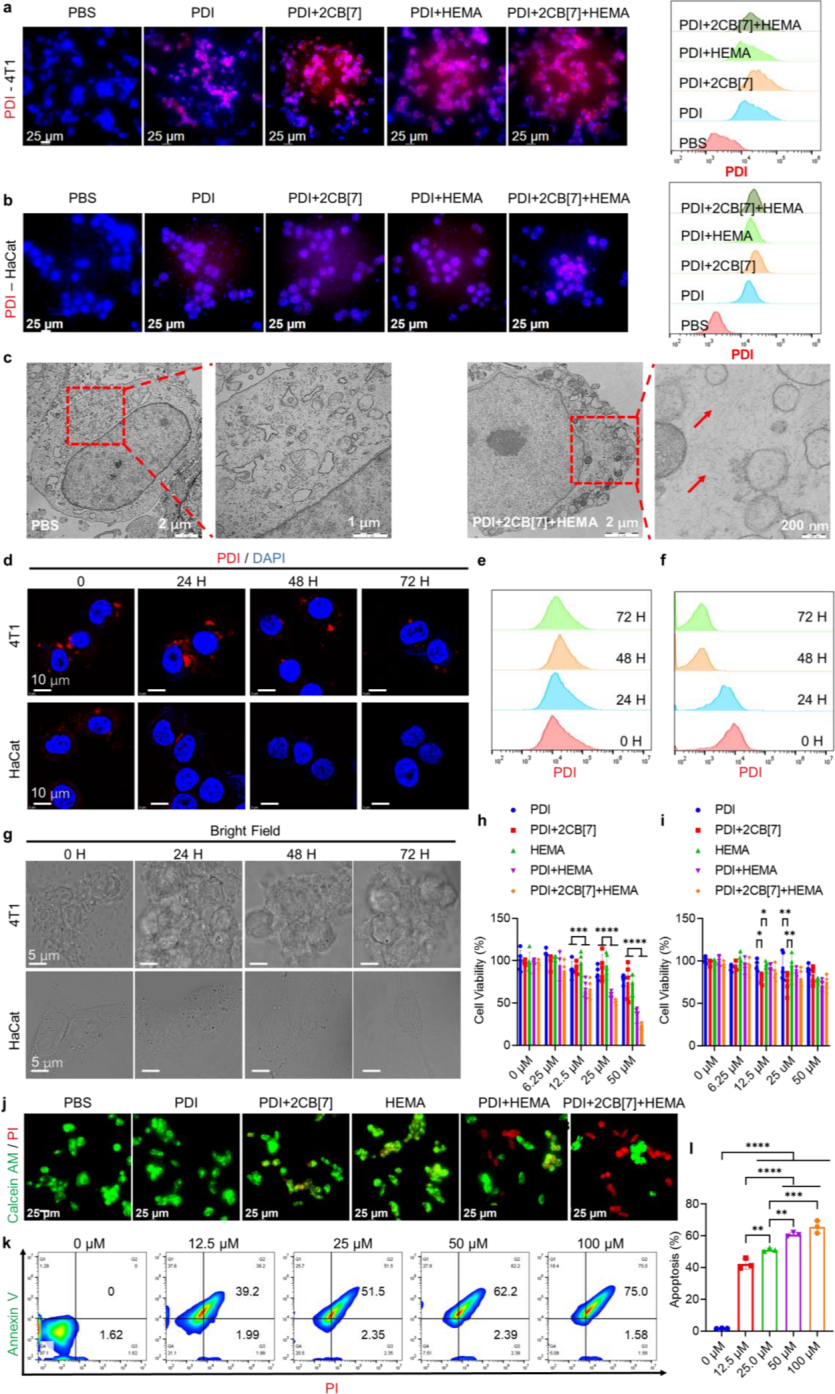
Investigation of intracellular polymerization and its cytotoxicity. (a) CLSM images and representative flow cytometry results of 4T1 cells incubated with PBS, PDI, PDI+2CB[7], PDI+HEMA, and PDI+2CB[7]+HEMA in a hypoxic environment. [CB[7]] = 2[PDI] = 2[HEMA] = 25.0 μM. DAPI, blue fluorescence. PDI, red fluorescence. (b) CLSM images and representative flow cytometry results of HaCat cells incubated with PBS, PDI, PDI+2CB[7], HEMA, PDI+HEMA, and PDI+2CB[7]+HEMA in a hypoxic environment. [CB[7]] = 2[PDI] = 2[HEMA] = 25.0 μM. (c) Biological transmission electron microscopy (Bio-TEM) images of 4T1 cells incubated with PBS or PDI+2CB[7]+HEMA. 4T1 cells were incubated in a hypoxic environment. [CB[7]] = 2[PDI] = 2[HEMA] = 25.0 μM. (d) CLSM images of 4T1 cells and HaCat cells incubated with PDI+2CB[7]+HEMA for different times. 4T1 cells were incubated in a hypoxic environment. [CB[7]] = 2[PDI] = 2[HEMA] = 25.0 μM. DAPI, blue fluorescence. PDI, red fluorescence. (e) Representative flow cytometry results of the fluorescence of 4T1 cells incubated with PDI+2CB[7]+HEMA in a hypoxic environment for different times. [CB[7]] = 2[PDI] = 2[HEMA] = 25.0 μM. (f) Representative flow cytometry results of the fluorescence of HaCat cells incubated with PDI+2CB[7]+HEMA for different times. [CB[7]] = 2[PDI] = 2[HEMA] = 25.0 μM. (g) CLSM images of 4T1 cells and HaCat cells incubated with PDI+2CB[7]+HEMA for different times. [CB[7]] = 2[PDI] = 2[HEMA] = 25.0 μM. Cell viability inhibition of different treatments on (h) 4T1 cells and (i) HaCat cells. 4T1 cells were incubated in a hypoxic environment. (j) CLSM images of Calcein AM/PI costaining of 4T1 cells after different treatments for 4 h. 4T1 cells were incubated in a hypoxic environment. [CB[7]] = 2[PDI] = 2[HEMA] = 25.0 μM. Calcein AM, green fluorescence; PI, red fluorescence. (k) Representative Annexin V-FITC/PI costaining flow cytometry results and (l) analytical results of 4T1 cells incubated with different treatments in a hypoxic environment. [PDI] = [HEMA] = 0, 12.5, 25, 50, 100.0 μM, [CB[7]] = 2[PDI]. One-way ANOVA and two-way ANOVA were utilized for statistical analysis. *P ≤ 0.05, **P ≤ 0.01, ***P ≤ 0.001, and ****P ≤ 0.0001 by one-way ANOVA and two-way ANOVA were applied to annotate statistical significance.

After displaying the enhanced free radical polymerization of PDI+2CB[7]+HEMA in a reducing condition, its possibility of biopolymerization in a living environment was further investigated. As shown in Figure S11, after coincubation for up to 3–4 h, the fluorescence intensity of 4T1 cells reached maximum changes and remained basically constant, suggesting the potential completion of intracellular polymerization. Thus, the incubation time was chosen to be 4 h in the subsequent cell experiments. 4T1 cells were incubated with PDI, PDI+2CB[7], PDI+HEMA, and PDI+2CB[7]+HEMA, respectively, under a hypoxic environment. HaCat cells cultured under a normoxic environment served as the control group. From the confocal images and flow cytometry results (Figures 2a,b and S12), stronger red fluorescence could be observed in both 4T1 and HaCat cells after incubation with PDI+2CB[7], in comparison to that of treatment with PDI or PDI+HEMA. This is mainly due to the fact that the complexation of CB[7] weakens the aggregation of PDI molecules, thereby increasing the fluorescence intensity to about 1.6 times for both cells. Meanwhile, different changes in the fluorescence emission behavior were observed in these two cell lines in the other groups. Larger clusters of fluorescence emission appeared in 4T1 cells (Figure 2a), attributed to the occurrence of polymerization reactions within 4T1 cells, which consumed part of PDI and generated supramolecular polymers. The fluorescence intensity of PDI+2CB[7]+HEMA was slightly weaker than that of PDI+2CB[7], while the fluorescence intensities of HaCat cells were almost the same. Moreover, biological transmission electron microscopy (Bio-TEM) was used to visually observe the occurrence of polymerization within cells. Obvious fibrous materials were observed inside 4T1 cells incubated with PDI+2CB[7]+HEMA in a hypoxic environment (Figure 2c). In contrast, HaCat cells treated with PDI+2CB[7]+HEMA exhibited no visible polymer fibers (Figures 2c and S13). These results demonstrated that PDI+2CB[7]+HEMA could selectively induce biopolymerization in 4T1 cells under a hypoxic environment. The chain termination processes of this intracellular polymerization were mainly due to the quenching of free radicals and PDI consumption. Since free radicals were quickly quenched in cells, the introduction of CB[7] could prolong the life of free radicals and increase the yield of free radicals to a certain extent; however, it still could not avoid the eventual quenching of free radicals in the complex cytoplasmic microenvironment. Moreover, the unsaturated bonds at the end of PDI participated in the polymerization process, and the consumption of PDI led to the end of the polymerization reaction. To determine the molecular weight of biosynthesized polymers in 4T1 cells, we isolated the polymer from 4T1 cells.^14^ Gel permeation chromatography (GPC) analysis showed a *M*_n_ of 15.5 kDa, and matrix-assisted laser desorption/ionization time-of-flight mass spectrometry analysis (MALDI-TOF) demonstrated the molecular ion intervals that are indicative of HEMA repeating units (Figure S14). Subsequently, the retention behavior of biosynthesized polymers inside of 4T1 cells was investigated. As shown in Figures 2d–f and S15, a stable red fluorescence intensity for up to 72 h was detected in 4T1 cells treated with PDI+2CB[7]+HEMA, while HaCat cells displayed a quick fluorescence decrease, indicating that the intracellular polymerization of small molecules could effectively prevent being expelled out.

Simultaneously, along with the increase in incubation time, 4T1 cells gradually changed their morphology, showing the potential that the polymeric fibers formed within 4T1 cells might affect their normal physiological functions (Figure 2g). However, HaCat cells still maintained their morphological integrity for a relatively long time. Thus, the impact of intracellular polymerization reactions on cell viability was investigated. As expected, 4T1 cells incubated with PDI+HEMA and PDI+2CB[7]+HEMA, respectively, in a hypoxic environment exhibited significant cell death, in comparison to cells treated individually with PDI, PDI+2CB[7] or HEMA, showing that the intracellular polymerization of PDI+HEMA or PDI+2CB[7]+HEMA caused cell damage (Figure 2h). At the same time, the cell death induced by PDI+2CB[7]+HEMA was stronger than that induced by PDI+HEMA, due to the improved polymerization after the addition of CB[7]. Meanwhile, HaCat cells were not affected by PDI+HEMA or PDI+2CB[7]+HEMA (Figure 2i). Similarly, the confocal images of Calcein AM/PI-costained 4T1 cells indicated significant cell death in 4T1 cells cultured with PDI+HEMA or PDI+2CB[7]+HEMA (Figure 2j), and a concentration-dependent effect was also measured on the apoptosis of 4T1 cells (Figures 2k and l). When 4T1 cells were treated with PDI+HEMA and PDI+2CB[7]+HEMA, respectively, at low concentrations, their internalized concentrations were quite low, leading to very little differences in cell viability. As the concentration increased, PDI+HEMA induced 4T1 cell death at a higher level, and the differences in cell viability between 4T1 cells treated with PDI+HEMA and those treated with PDI+2CB[7]+HEMA became larger and both exhibited a dose-dependent manner. This result suggested that CB[7] complexation contributed to the tumor growth inhibition effect of PDI+HEMA. Indeed, the intracellular polymerization strategy herein mainly relies on the initiation of radical anions formed by PDI. Since radical anions are extremely sensitive and are rapidly quenched in the complex microenvironment inside cells, the introduction of CB[7] could significantly reduce the LUMO and HOMO energies of PDI molecules, weaken the close stacking of PDI aromatic cores, and inhibit the dimerization and quenching of PDI radical anions, thereby increasing the yield of free radicals in aqueous solutions.^26−28^ Collectively, this system could selectively undergo polymerization reactions in tumor cells under hypoxic conditions, thereby causing severe cytotoxicity.

**Figure 3:**
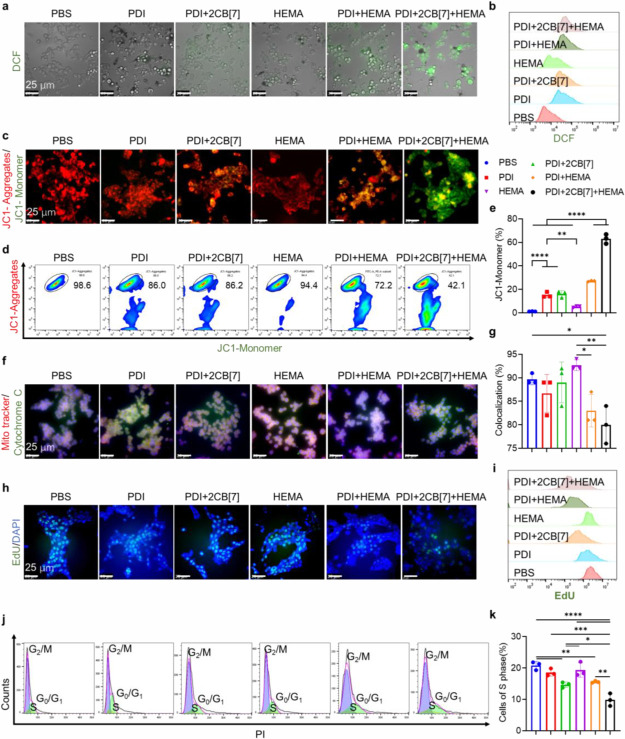
Impact of intracellular polymerization reactions on cellular function. (a) CLSM images of ROS generation in 4T1 cells incubated with different treatments, as indicated by the green fluorescence of 2′,7′-dichlorofluorescein (DCF) that was oxidized from H_2_DCFDA by ROS. [CB[7]] = 2[PDI] = 2[HEMA] = 25.0 μM. (b) Representative ROS staining flow cytometry results of 4T1 cells treated with different treatments. [CB[7]] = 2[PDI] = 2[HEMA] = 25.0 μM. (c) CLSM images of mitochondrial membrane potential measurement in 4T1 cells with a JC-1 assay kit. [CB[7]] = 2[PDI] = 2[HEMA] = 25.0 μM. PDI, red fluorescence. Mitochondria tracker, green fluorescence. (d, e) Representative flow cytometry results of mitochondrial membrane potential measurement of 4T1 cells with the JC-1 assay kit. [CB[7]] = 2[PDI] = 2[HEMA] = 25.0 μM. (f) Release of cytochrome c from mitochondria in cells. Mitochondria, red fluorescence. Cytochrome *c*, green fluorescence. (g) Quantitative results of cytochrome *c* released from mitochondria in cells. [CB[7]] = 2[PDI] = 2[HEMA] = 25.0 μM. (h) Cell proliferation study using an EdU staining assay. CLSM images of 4T1 cells incubated with different treatments. [PDI] = [HEMA] = 12.5 μM; [CB[7]] = 25.0 μM. DAPI, blue fluorescence. EdU, green fluorescence. (i) Representative EdU staining flow cytometry results of 4T1 cells incubated with different treatments. [CB[7]] = 2[PDI] = 2[HEMA] = 25.0 μM. (j), (k) Cell cycle was investigated by treating the 4T1 cells with different treatments. [CB[7]] = 2[PDI] = 2[HEMA] = 25.0 μM. Treated cells were fixed with 70% ethanol overnight, treated with RNase A, stained by PI, and analyzed by flow cytometry. One-way ANOVA was utilized for statistical analysis. *P ≤ 0.05, **P ≤ 0.01, ***P ≤ 0.001, and ****P ≤ 0.0001 by one-way ANOVA were applied to annotate statistical significance.

Due to the cell death and damage induced by intracellular free radical polymerization, our research interest has been drawn to the mechanism behind this process. First, 2′,7′-dichlorodihydrofluorescein diacetate (H_2_DCFDA) was used to indicate the generation of reactive oxygen species (ROS), and all 4T1 cells treated with PDI, PDI+2CB[7], and HEMA showed low green fluorescence intensity, determined by confocal imaging and flow cytometry (Figure 3a). In contrast, the fluorescence intensity was increased in PDI+HEMA and PDI+2CB[7]+HEMA-treated 4T1 cells, suggesting a higher ROS level. Furthermore, quantitative analysis revealed that the ROS content in PDI+2CB[7]+HEMA-treated 4T1 cells was 1.25 times higher than that in cells incubated with PDI+HEMA (Figures 3b and S16), indicating that intracellular polymerization mediated by supramolecular free radicals induced ROS overproduction. Mitochondria, as the energy factory of cells, play a crucial role in maintaining the normal activities and functions of cells and are easily induced to dysfunction by overproduced ROS. Thus, the change in mitochondrial function before and after intracellular polymerization aroused our research interest. The mitochondrial membrane potential (MMP) was assessed by the JC-1 kit in 4T1 cells after different treatments. As shown in Figure 3c, the green fluorescence of the JC1 monomer was significantly enhanced, while the red fluorescence of JC1 aggregation was weakened in PDI+2CB[7]+HEMA-treated cells, exhibiting that the cellular MMP level was significantly reduced. However, the MMP of 4T1 cells treated with PDI, PDI+2CB[7], HEMA, and PDI+HEMA, respectively, exhibited negligible changes, comparable to the PBS group. Flow cytometry analysis further confirmed that PDI+2CB[7]+HEMA treatment significantly decreased the MMP, with the JC1 monomer accounting for the majority (Figure 3d,e). Moreover, 4T1 cells treated with PDI+2CB[7]+HEMA showed a high early apoptosis rate and inhibited ATP production, which was always accompanied by a MMP decrease (Figure S17). Fluorescence colocalization intensity between mitochondrial (red) and cytochrome *c* (green) was reduced in PDI+2CB[7]+HEMA-treated 4T1 cells in comparison to other treated groups, exhibiting the leakage of cytochrome c from mitochondria and mitochondria damage (Figure 3f,g).

In addition to the physiological functions of organelles, the EdU staining assay was used to investigate the effect of intracellular polymerization on cell cycle. As shown in Figure 3h, 4T1 cells treated with PDI+HEMA showed a slight green fluorescence intensity, while treatments with PDI, PDI+2CB[7], or HEMA induced no significant changes. In contrast, the fluorescence intensity significantly decreased in 4T1 cells incubated with PDI+2CB[7]+HEMA, indicating that highly efficient intracellular polymerization induced cell cycle arrest. Quantitative analysis showed a significant decrease in the synthesized DNA content of PDI+2CB[7]+HEMA-treated 4T1 cells, down to 40.8% of the control group (Figures 3i and S18). Interestingly, after receiving treatments for 4 h, the G_0_/G_1_ phases between all treated and untreated cells were almost the same (Figures 3j and S19a); however, 4T1 cells treated with PDI+2CB[7]+HEMA specifically decreased the accumulation of the S phase (6% of cells were in the S phase, while untreated cells accounted for 19%), indicating that the synthesis of intracellular DNA was obviously inhibited by intracellular polymerization (Figure 3j,k). Similarly, G_2_/M phase cell cycle arrest was also observed in 4T1 cells treated with PDI+2CB[7]+HEMA (from 34.9 to 48.9%), and the other treated groups showed no significant changes (Figures 3k and S19b). This result indicated that intracellular polymerization started to affect cell cycle after passing through the G_2_/M transition point, prolonging the time to enter the division phase and resulting in an extension of the entire cell cycle and slow proliferation. Collectively, supramolecular free-radical-mediated intracellular polymerization induced ROS overexpression and mitochondrial dysfunction, thereby leading to cell apoptosis and cell cycle arrest.

**Figure 4:**
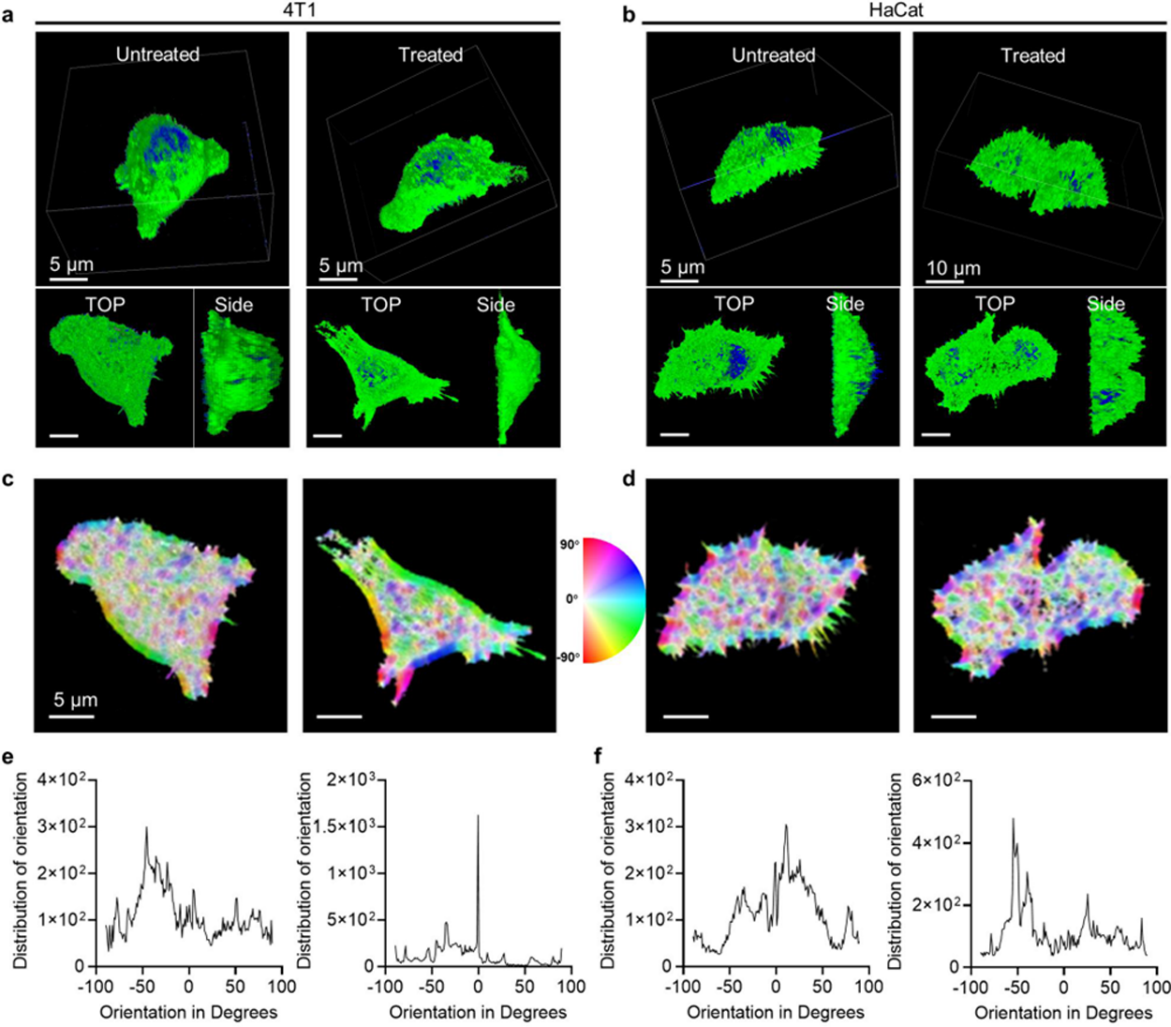
Changes in actin cytoskeleton organization in intracellularly polymerized cells. (a) 4T1 cells treated with and without PDI+2CB[7]+HEMA. (b) HaCat cells treated with and without PDI+2CB[7]+HEMA were stained for actin filaments (F-actin, after removal of cellular membranes). [CB[7]] = 2[PDI] = 2 [HEMA] = 25.0 μM. Corresponding orientation plots for actin staining of (c) 4T1 cells and (d) HaCat cells, where the different colors indicate different orientations of actin filaments per the given color map. The microdomain distributions for actin filament orientations of (e) 4T1 cells and (f) HaCat cells (as measured by ImageJ).

Intracellular polymerization provided an artificial network in the cytoplasm and would interfere with the actin structure and increase the viscosity and mechanical properties.^29−31^ To better understand the effect of intracellular polymerization on the cytoskeleton, the change in the cytoplasmic F-actin was analyzed in PDI+2CB[7]+HEMA-treated 4T1 cells. Three-dimensional fluorescence imaging showed that F-actin in untreated cells presented a long-range ordered and locally disordered filamentous structure, and the shape was related to the orthoradial actin distribution. The small adhesion complexes were mostly distributed at the cell edges and wrapped around the entire cell (Figure 4a,b). However, intracellular polymerization in PDI+2CB[7]+HEMA cells showed a local uniform and orderly distribution of F-actin and a longer polarized morphology (Figure 4a). Since the polymerization reaction could not be induced in HaCat cells incubated with PDI+2CB[7]+HEMA, nearly no obvious change of the F-actin structure was detected after treatment with or without PDI+2CB[7]+HEMA (Figure 4b). Furthermore, the color orientation plots were used to quantify the local angles of actin filaments in treated cells. As shown in Figures 4 c–f, the order of actin in treated 4T1 cells increased significantly in comparison to that of untreated 4T1 cells, suggesting that intracellular polymerization interfered with the actin network, thereby destroying the mechanical support of the cell. In contrast, the fluorescence intensity and the distribution of orientation were the same for HaCat cells treated with or without PDI+2CB[7]+HEMA. Therefore, these results revealed that efficient intracellular polymerization also changed the order distribution of F-actin and actin, leading to cytoskeleton damage.

**Figure 5:**
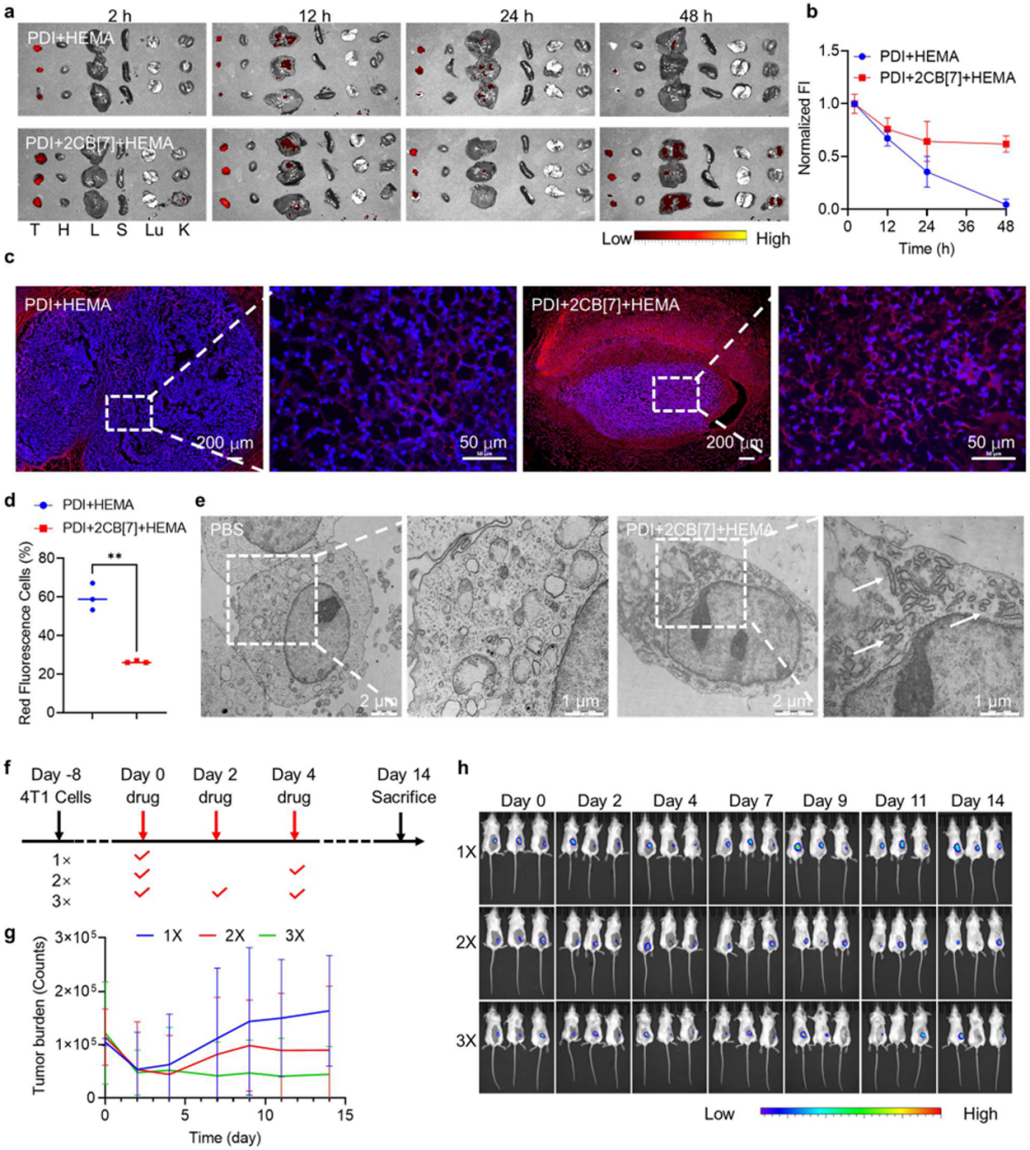
Intracellular polymerization led to tumor suppression. (a) 4T1 tumor-bearing mice were intratumorally injected with PDI+HEMA or PDI+2CB[7]+HEMA, [CB[7]] = 2[PDI] = 2 [HEMA] = 50.0 μM, 100 μL. After administration for different durations (2, 12, 24, and 48 h), ex vivo fluorescence imaging was conducted on tumor (T), heart (H), liver (L), spleen (S), lungs (Lu), and kidneys (K) from treated mice. (b) Changes in relative fluorescence intensity (FI) in tumors from the treated groups. (c) Fluorescence imaging of tumors in the treated groups. DAPI (blue), PDI (red). (d) Semiquantitative analysis of the red fluorescent cells. **P ≤ 0.01 by Student’s *t-*test was applied to annotate statistical significance. (e) Bio-TEM images of tumors in the treated groups. (f) Schematic illustration of the antitumor experiment in mice. (g) Graph of tumor burden across time of control and treated mice. (h) IVIS bioluminescence images of 4T1 tumor growth (received 1–3 times injection of PDI+2CB[7]+HEMA, [CB[7]] = 2[PDI] = 2 [HEMA] = 50.0 μM, 100 μL).

Previous evidence verified that the mixture system (PDI+2CB[7]+HEMA) could polymerize in 4T1cells under a hypoxic environment via hypoxia-induced free radicals and provide the potential application for selectively in situ polymerization in the tumor site. Herein, BALB/c mice were subcutaneously injected with 10^6^ of 4T1 cells into the right hind leg to establish a subcutaneous tumor model after administration for 8 days. Tumor-bearing mice were randomly divided into three groups (*n* = 12) and intratumorally administered with PDI+HEMA and PDI+2CB[7]+HEMA at a dose of 25.0 μM, respectively. The heart, liver, spleen, lung, and kidney were collected at different time points after administration (2, 12, 24, and 48 h) for ex vivo fluorescence imaging. Throughout the entire experimental period (up to 48 h), PDI+2CB[7]+HEMA-treated mice maintained the fluorescence intensity at a high level in the tumor, consistently stronger than that of other organs, exhibiting great tumor retention. This result should be attributed to supramolecular free-radical-mediated intracellular polymerization in response to the hypoxic tumor microenvironment. However, the fluorescence intensity of tumor tissue quickly decreased in PDI+HEMA-treated mice, and the fluorescence intensity in the liver and kidney gradually increased (Figure 5a,b), demonstrating that the addition of CB[7] enhanced the polymerization efficiency in tumor tissue and prolonged the tumor retention time. This phenomenon was beneficial because supramolecular complexation between PDI and CB[7] enhanced and stabilized free radical production (Figure 2a), thereby facilitating polymerization in tumor tissue. At the end of the experiment, the mice were euthanized, and the tumors were removed for section and fluorescence imaging (Figure 5c,d). Compared with the mice treated with PDI+HEMA (26.4%), PDI+2CB[7]+HEMA-treated mice showed 59.8% red fluorescence areas throughout the entire section. The enlarged fluorescence image accurately confirmed that the fluorescence signal was located in the cytoplasm of 4T1 cells, further confirming that the high tumor retention effect of PDI+2CB[7]+HEMA was attributed to intracellular polymerization triggered by the reducing and hypoxic microenvironments inside 4T1 cells. Moreover, to directly demonstrate the occurrence of polymerization reactions in 4T1 cells, bio-TEM analysis was performed on tumor sections from PDI+2CB[7]+HEMA-treated mice. Compared with untreated mice, a large number of polymer fiber materials could be clearly observed in the cytoplasm, demonstrating the formation of intracellular polymers in tumor tissue (Figure 5e). The polymer morphology was more obvious than that in Figure 2c, probably due to the higher treatment concentration of PDI+2CB[7]+HEMA.

Regarding the cell damage and cell death induced by intracellular polymerization, the antitumor effect of PDI+2CB[7]+HEMA was investigated. BALB/c mice were subcutaneously injected with 100 μL of luciferase 4T1 cells (10^7^ cells/mL) into the right hind leg to establish a fluorescent tumor model, and the administration route, dosage, and schedule are shown in Figure 5f. Tumor burden was monitored by bioluminescence from IVIS spectral imaging and quantified in counts (Figure 5g,h). These results showed that the tumor growth of mice treated with one or two doses of PDI+2CB[7]+HEMA was significantly inhibited during the initiation phase of drug administration, but the tumor grew rapidly again. However, one out of three mice treated with three doses completely cleared the tumor. This result demonstrated that intracellular polymerization initiated in tumor tissue provides a nondrug and precision antitumor strategy.

**Figure 6:**
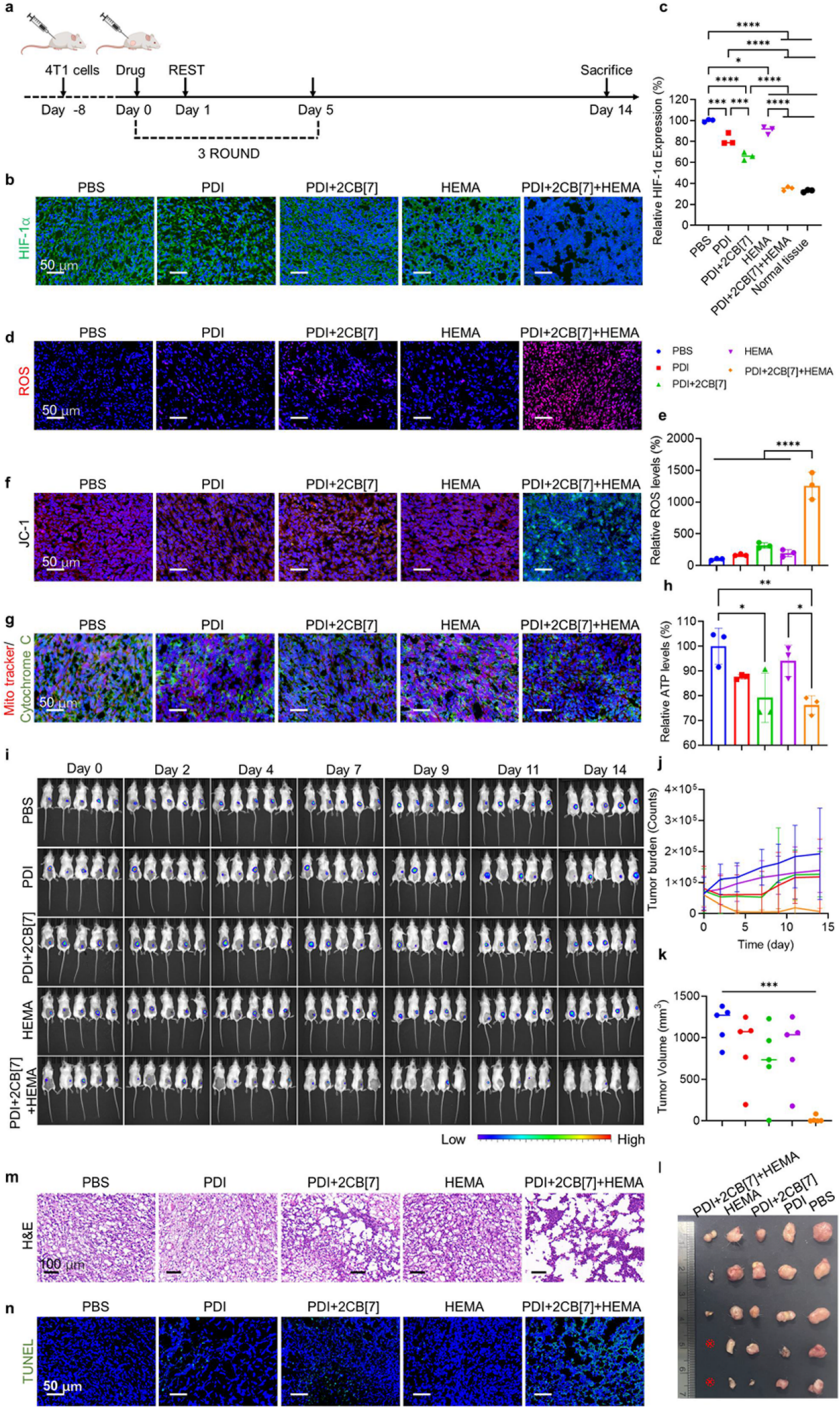
Tumor therapeutic effects induced by intracellular polymerization. (a) Schematic illustration of the antitumor experiment in mice. (b) Fluorescence images of tumors from mice treated with PBS, PDI, PDI+2CB[7], HEMA, and PDI+2CB[7]+HEMA. Sections were stained with DAPI (blue) and HIF-1α (green). (c) HIF-1α expression of tumors after different treatments. (d) Sections were stained with DAPI (blue) and ROS (red). (e) Semiquantitative analysis of ROS was carried out through ImageJ. (f) Sections were stained with DAPI (blue) and JC-1 (red and green). (g) Sections were stained with DAPI (blue), mitochondria tracker (red), and cytochrome c (green). (h) Relative ATP levels in the different treatments’ groups. (i) IVIS bioluminescence images of 4T1 tumor growth. (j) Graph of tumor burden (counts) and (k) tumor growth curve across time of mice treated with PBS, PDI, PDI+2CB[7], HEMA, and PDI+2CB[7]+HEMA. Mice received 3 treatments. (l) At the end of 15 days of observation, the mice were euthanized, and the tumors were removed and photographed. (m) H&E and (n) TUNEL photos of the tumors of mice after different treatments. One-way ANOVA was utilized for statistical analysis. *P ≤ 0.05, **P ≤ 0.01, ***P ≤ 0.001, and ****P ≤ 0.0001 by one-way ANOVA were applied to annotate statistical significance.

To investigate the in vivo antitumor mechanism of PDI+2CB[7]+HEMA, tumor-bearing mice were randomly divided into five groups and intratumorally administered with PBS, PDI, PDI+2CB[7], HEMA, and PDI+2CB[7]+HEMA, respectively, at a dose of 25.0 μM once every 2 days for a total of three doses (Figure 6a). On the third day after the last dose, one batch of the mice was euthanized, and the tumor tissues were collected for paraffin embedding, sectioning, and staining analysis. Immunohistological staining showed that the HIF-1α expression in tumors from PDI+2CB[7]+HEMA-treated mice was significantly reduced by 60% in comparison to that of PBS-treated mice, close to that of normal tissue (Figures 6b,c, and S20). This phenomenon might be attributed to the fact that the intracellular polymerization reaction caused extensive cell damage, thereby inhibiting oxygen consumption. DCFH-DA staining on the tumor section showed the highest green fluorescence intensity in PDI+2CB[7]+HEMA-treated mice (Figure 6d). Quantitative results exhibited that the ROS content in the tumor section of mice that underwent polymerization was 13 times higher than that of PBS-treated mice (Figure 6e), proving that efficient intracellular polymerization caused great ROS production and potential damage to tumor tissue. Since a large intracellular accumulation of ROS could seriously damage the mitochondrial function, the changes in MMP in tumor tissue were also investigated. Tumor section from PDI+2CB[7]+HEMA-treated mice showed a strong green fluorescence signal related to the JC1 monomer. In contrast, the other treated groups showed a distinct red signal associated with JC1 aggregates (Figure 6f). These results indicated that intracellular polymerization could induce a decrease in MMP values, leading to mitochondrial dysfunction and apoptosis. In addition, ROS in cells also caused cytochrome c to be massively released from mitochondria, as evidenced by the decrease in the fluorescence colocalization of mitochondria (red) and cytochrome c (green) (Figure 6g). Subsequently, the cell’s energy supply was greatly reduced (Figure 6h), further leading to the antitumor efficacy of PDI+2CB[7]+HEMA.

Afterward, the tumor growth of mice receiving different treatments was imaged by the IVIS system for 15 days, and the tumors from PBS-treated mice and HEMA-treated mice grew rapidly. The tumor growth of mice in the PDI and PDI+2CB[7] group was inhibited to a certain extent at the initial administration stage but eventually grew rapidly again. In contrast, PDI+2CB[7]+HEMA treatment significantly inhibited the tumor growth, and two out of five mice completely eliminated tumors (Figure 6i,j). On the last day of observation, all mice were euthanized, and tumors were removed for photography and histological analysis. As shown in Figure 6k,l, PDI+2CB[7]+HEMA-treated mice had the smallest tumor volume, supporting hypoxic-initiating intracellular polymerization in tumor tissue precisely and effectively inhibited tumor growth. Hematoxylin and eosin (H&E) staining showed that the cells of tumor tissue were dense in mice treated with PBS, PDI, and HEMA (Figure 6m), indicating no tumor damage after these treatments. However, the tumor tissue was loose, and a large number of lesion areas was observed in mice-treated PDI+2CB[7] and PDI+2CB[7]+HEMA. As expected, the tumor tissue of PDI+2CB[7]+HEMA-treated mice was the most severely damaged. In addition, as shown in the TUNEL staining result of Figure 6n, the green fluorescence in the tumor section from PDI+2CB[7]+HEMA-treated mice was the strongest, referring to the most apoptotic cells. Thus, hypoxia-initiated intracellular polymerization achieved excellent antitumor efficacy.

Finally, to further justify the use of intratumoral injection, we systematically evaluated the biosafety of this approach, and the results are shown in Figure S21. The tumor-bearing mice were randomly divided into five groups, and the organs (heart, liver, spleen, lung, and kidney) and blood were collected after administration for 3 days for biosafety evaluation. H&E staining on the heart, liver, spleen, lungs, and kidneys of mice in all treated mice showed negligible physiological changes and tissue damage (Figure S21a), indicating again that the selective intracellular polymerization of PDI+2CB[7]+HEMA has good biocompatibility and biosafety. Furthermore, the counts of blood WBC, RBC, HGB, HCT, MCV, MCH, MCHC, RDW, PLT, and lymph in the blood of the PDI+2CB[7]+HEMA-treated mice were nearly identical to those of the healthy mice. The liver and kidney function biomarkers, ALT, AST, TP, CREA, and BUN, also maintained in the normal range, proving a good safety profile for utilizing hypoxic conditions to initiate intracellular polymerization for effective cancer treatment via intratumoral injections (Figure S21b).

## Conclusions

We have developed a method to induce in situ free radical polymerization within tumor cells using endogenous cellular stimuli, resulting in the formation of durable polymers that exhibit therapeutic efficacy in a mouse subcutaneous tumor model. Tumor cells, characterized by significant hypoxia, accumulate high levels of reducing substances, triggering the formation of PDI+2CB[7] supramolecular free radicals. These radicals initiate a polymerization reaction with HEMA, generating substantial polymer deposits within tumor cells. The prolonged presence of these polymers effectively inhibits cell growth and induces apoptosis.

Furthermore, intracellular polymerization leads to the accumulation of ROS, disrupting mitochondrial function and energy supply and causing cell cycle arrest. Additionally, the biosynthesized polymers disrupt the cytoskeleton’s dynamic balance, significantly impairing tumor cell migration. In vivo evaluations confirm the sustained intracellular polymerization and extended retention within tumor tissues, contributing to potent antitumor effects. Throughout treatment, this strategy demonstrates excellent biocompatibility and effectively alleviates tumor hypoxia, achieving safe and precise antitumor therapy goals.

Compared with existing intracellular free radical polymerization methods, our approach offers several advantages. It introduces a novel strategy for selectively aggregating polymers within tumor cells without pharmacological agents. The polymerization reaction relies solely on endogenous tumor cell stimuli, enhancing safety and reaction efficiency. The supramolecular host–guest strategy stabilizes intracellular free radicals, improving yield and accelerating polymerization reactions within tumor cells.

Further studies are necessary to elucidate clearance pathways and pharmacokinetics of PDI+2CB[7]+HEMA in vivo, crucial for advancing clinical applications. While demonstrating excellent selectivity between tumors and normal tissues, the current strategy would require intratumoral injection due to the lack of tumor-targeting groups during delivery. Future research should focus on developing next-generation drugs with enhanced tumor-targeting capabilities and specificity.

In conclusion, our approach harnesses endogenous biological stimuli to rapidly advance precision medicine through selective intracellular polymerization chemistry, offering a promising path for future cancer therapeutics development.

## Supporting Information Available

Materials, instruments, experimental section, animal ethical statement, synthetic routes, NMR spectra, high-resolution mass spectra, UV–vis spectra, fluorescence emission spectra, flow cytometry results, Bio-TEM images, fluorescence images of normal tissue, H&E staining images, and blood analysis results (PDF)

## Abbreviations

PDI: perylene diimide derivative
CB[7]: cucurbit[7]uril
HEMA: 2-hydroxyethyl methacrylate
HRMS: high-resolution mass spectrometry
COSY: correlation spectroscopy
Na_2_S_2_O_4_: sodium dithionite
EPR: electron paramagnetic resonance
ROS: reactive oxygen species
H_2_DCFDA: 2′,7′-dichlorodihydrofluorescein diacetate
DCF: 2′,7′-dichlorofluorescein
SEM: scanning electron microscopy
Bio-TEM: biological transmission electron microscopy
