# Supplementary material for: Hypoxia-Initiated Supramolecular Free Radicals Induce Intracellular Polymerization for Precision Tumor Therapy
Source: J Am Chem Soc. 2025 Jan 13;147(4):3488–99. doi: 10.1021/jacs.4c14847 (PMC11783515; doi:10.1021/jacs.4c14847)
Supplement: Supplementary file 1 — ja4c14847_si_001.pdf [file ja4c14847_si_001.pdf]

Supporting information

Hypoxia-initiated supramolecular free radicals induce intracellular polymerization for precision tumor therapy

Mian Tang[1], Zhiqing Yang[1], Xingchen Tang[2], He Ma[2], Beibei Xie[1], Jiang-Fei Xu[2], Cheng Gao[3]*, David Bardelang[4], and Ruibing Wang[1]*

[1] State Key Laboratory of Quality Research in Chinese Medicine, Institute of Chinese Medical Sciences, and MoE Frontiers Science Center for Precision Oncology, University of Macau, Taipa, Macau SAR 999078, China

[2] Key Lab of Organic Optoelectronics & Molecular Engineering, Department of Chemistry, Tsinghua University, Beijing 100084, China

[3] School of Pharmacy, Shenzhen University Medical School, Shenzhen University, Shenzhen, 518055, PR China

[4] CNRS, ICR, AMUtech, Aix-Marseille University, Marseille F-13397, France

E-mail: chenggao@szu.edu.cn; rwang@um.edu.mo

Table of contents:

1. **General Information**
2. **Experimental Procedures**

   2.1 Animal Ethical Statement

   2.2 Cell viability assay

   2.3 Inhibition of tumor growth experiments

   2.4 The imaging parameters for the IVIS.

   2.5 Safety evaluation
3. **Results and Discussion**

   3.1 Synthesis of PDI

   3.2 Characterization of PDI and PDI+2CB[7]

   3.3 The fluorescence intensity of 4T1 cells after different incubation times.

   3.4 Relative fluorescence intensity of 4T1 cells and HaCat cells after different treatments.

   3.5 Bio-TEM images of HaCat cells after different treatments.

   3.6 Characterization of intracellular polymers.

   3.7 Relative fluorescence intensity of 4T1 cells and HaCat cells after different incubation time.

   3.8 Relative ROS levels in4T1 cells after different treatments.

   3.9 Relative ATP levels in4T1 cells after different treatments.

   3.10 Relative Edu intensity in 4T1 cells after different treatments.

   3.11 The cell cycle of 4T1 cells after different treatments.

   3.12 The HIF-1α level of normal tissue.

   3.13 The safety evaluation.
4. **References**

1. General Information

All chemicals were commercial reagent grade and used without further purification, unless otherwise stated. NMR spectra were recorded on Bruker 600 MHz instrument, and chemical shifts were recorded in parts per million (ppm). SEM images were acquired by Zeiss Gemini 300. The samples were prepared by placing a drop of solution onto a silicon wafer and air-dried. Bio-TEM images were acquired by HITACHI H-7650. UV-vis absorption spectra were recorded on UV-vis spectrophotometer (DR6000, HACH) in a quartz cell (light path 10 mm) at 25 °C and 37 °C with a PTC-348WI temperature controller. Fluorescence spectra were recorded on Thermo Scientific Lumina Fluorescence Spectrometer (THERMO-LUMINA) in a quartz cell (light path 10 mm) at 25 °C. CytoFLXE S Flow Cytometer (Beckman Coulter) was used to flow cytometry analysis experiments. Confocal laser scanning microscopy (CLSM, Zeiss LSM710) was used at cell imaging experiment. Multimode microplate reader (FlexStation3) was used to evaluate the cell proliferation and cytotoxicity assay kit (cck-8 assay) results. All in vitro experiments were carried out independently for more than or equal to three times. The data were shown as mean value ± the standard deviation of independent experiments. In vivo mouse experiments were set as n = 5 for each group. One-way ANOVA and students' t test were utilized for statistical analysis. Value of $*P \leqslant 0.05$, $**P \leqslant 0.01$, $***P \leqslant 0.001$ and $****P \leqslant 0.0001$were applied to annotate statistical significance.

2. Experimental Procedures

2.1 Animal Ethical Statement

All animal procedures were approved by the Animal Ethics Committee, Medical School, Shenzhen University (IACUC-202400065), and were conducted in accordance with the Animal Management Rules of the Ministry of Health of the PR China.

2.2 Cell viability assay

To investigate the toxicity of intracellular polymerization, cells were cultured in 96-well plates in 1640 medium or DMEM medium containing 10% FBS for 24 h, and then the corresponding samples were added into the wells, the cells were further cultured for 4 h. Then, the cell growth was calculated by cell proliferation and cytotoxicity assay kit (cck-8 assay).

2.3 Inhibition of tumor growth experiments

To examine the in vivo anticancer ability, 6-week-old female BALB/c mice were subcutaneously injected 100 μL of Luciferase 4T1 cells (10^7 cells/mL) into the right hind leg to establish a subcutaneous tumor model. After injection for 8 days, the volume of the tumor was about 80 mm^3, the tumor burden mice were blindly and randomly separated into five groups (n = 5) and injected with PBS (100 μL), PDI (25.0 μM, 100 μL), PDI+2CB[7] ([CB[7]] = 2[PDI] = 50.0 μM, 100 μL), HEMA (25.0 μM, 100 μL), and PDI+2CB[7]+HEMA ([CB[7]] = 2[PDI] = 2[HEMA] = 50.0 μM, 100 μL) (once per 2 days), respectively. When injecting intratumorally, the tumor was divided into three equal points, and then injected sequentially. The syringe was at an angle of about 45° to the animal's body, and the needle was inserted avoiding the subcutaneous blood vessels, then the drug solution was slowly pushed into the tumor.

2.4 The imaging parameters for the IVIS.

200 μL (15 mg mL^{-1}) of sodium fluorescein was injected intraperitoneally into the mice before in vivo imaging.

Mode: luminescence

Exposure time: 1 s

Binning: 4

Emission: 570 nm

2.5 Safety evaluation

At experimental endpoint of anti-tumor treatment, organs (heart, liver, spleen, lung and

kidney) and tumor collected from all groups for pathologic analysis on potential side effects and whole blood and serum were collected from PBS and PDI+2CB[7]+HEMA groups. The levels of serum ALT, AST, BUN, CREA, TP and UREA in all treated mice were quantified by biochemical analysis. In addition, histological analysis was conducted on heart, liver, spleen, lung and kidney. The content of WBC, RBC, HGB, HCT, MCV, MCH, MCHC, RDW, PLT and Lym# was measured.

3. Results and Discussion

3.1 Synthesis of PDI

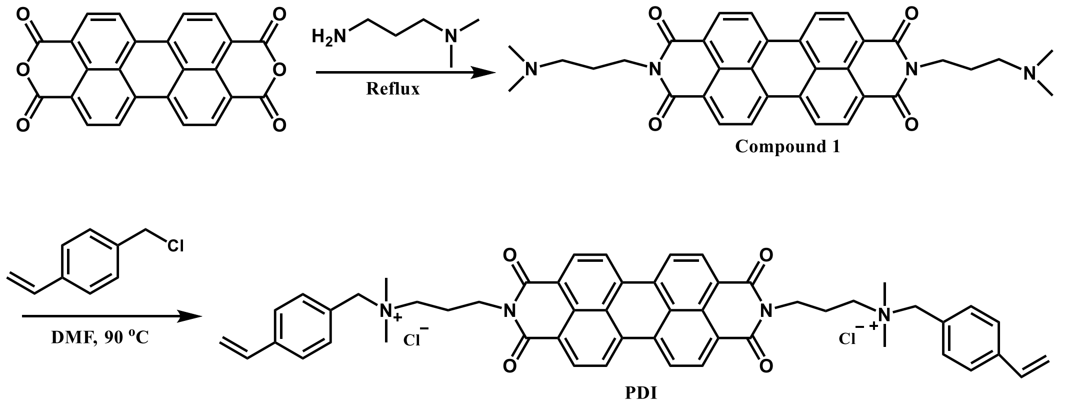

Figure S1: Synthetic route of Compound 1 and PDI.

Compound 1 was synthesized according to the reported procedures.[1] 0.1 g Compound 1 and 1.0 g 4-vinylbenzyl chloride were mixed in 20 mL DMF. The mixture was then stirred at 90°C for 12 h. The dark red solid precipitate (PDI) was filtered directly and washed by acetonitrile and diethyl ether for three times. The yield was 95 %.

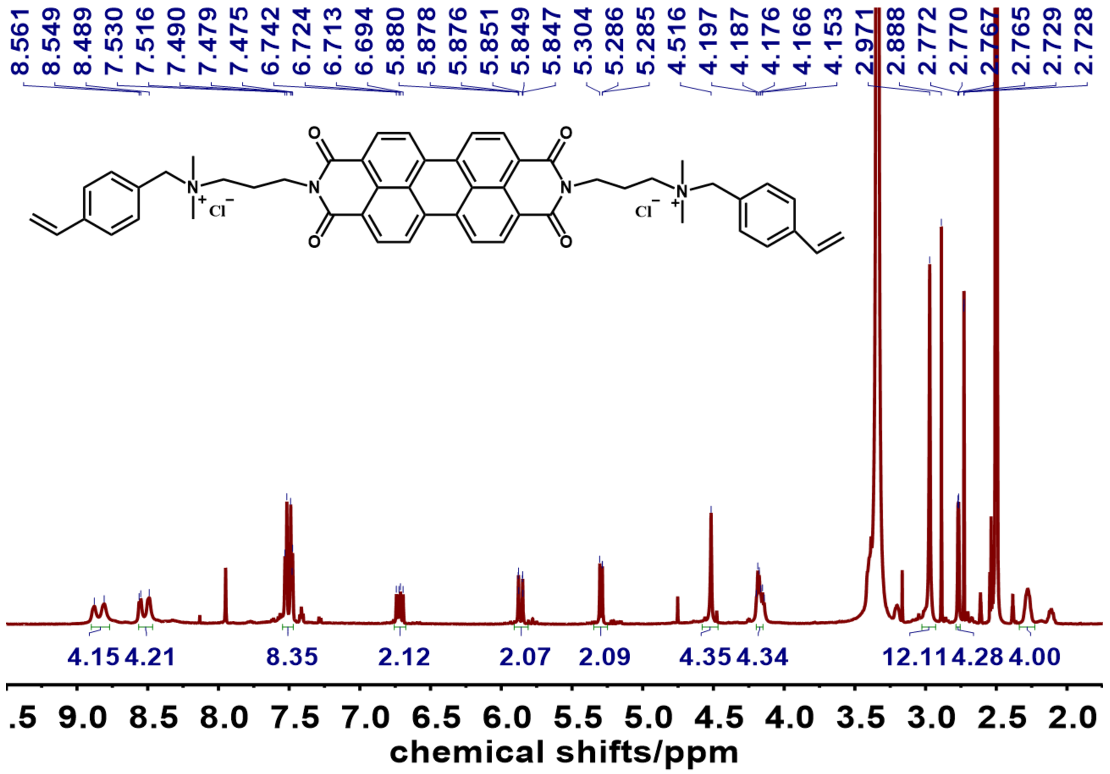

Figure S2: ^{1}H NMR spectrum (600 MHz, DMSO-d_6, 25 °C) of PDI.

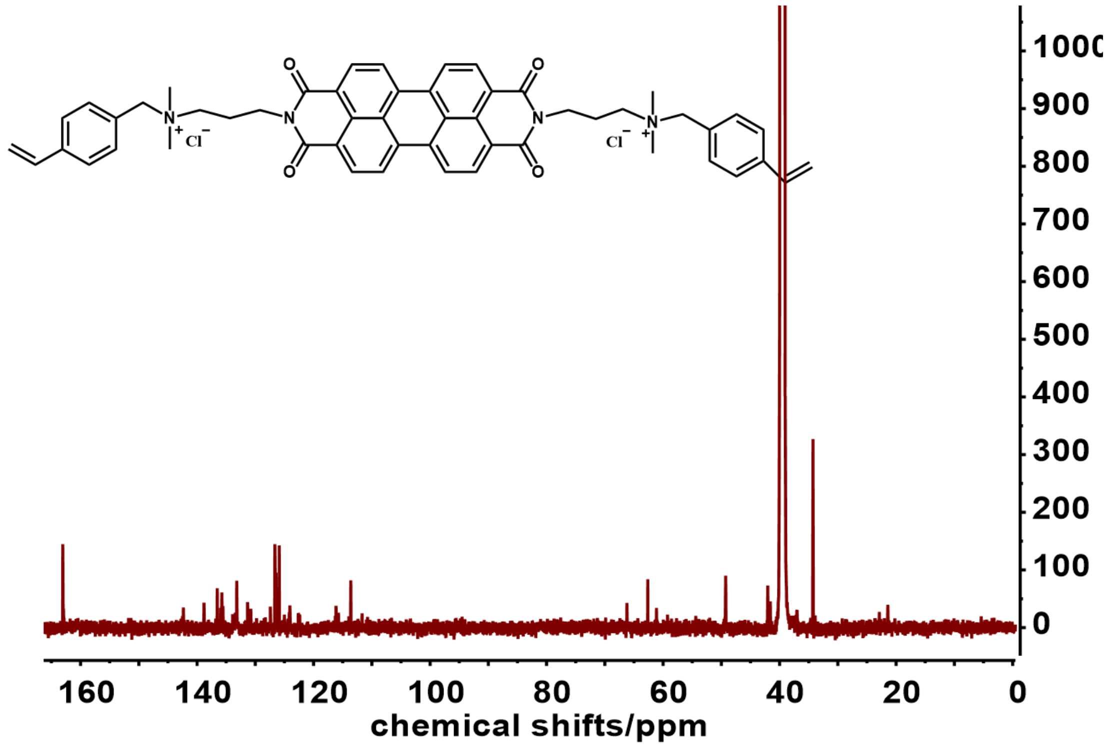

Figure S3: ^{13}C NMR spectrum (600 MHz, DMSO-d_6, 25 °C) of PDI.

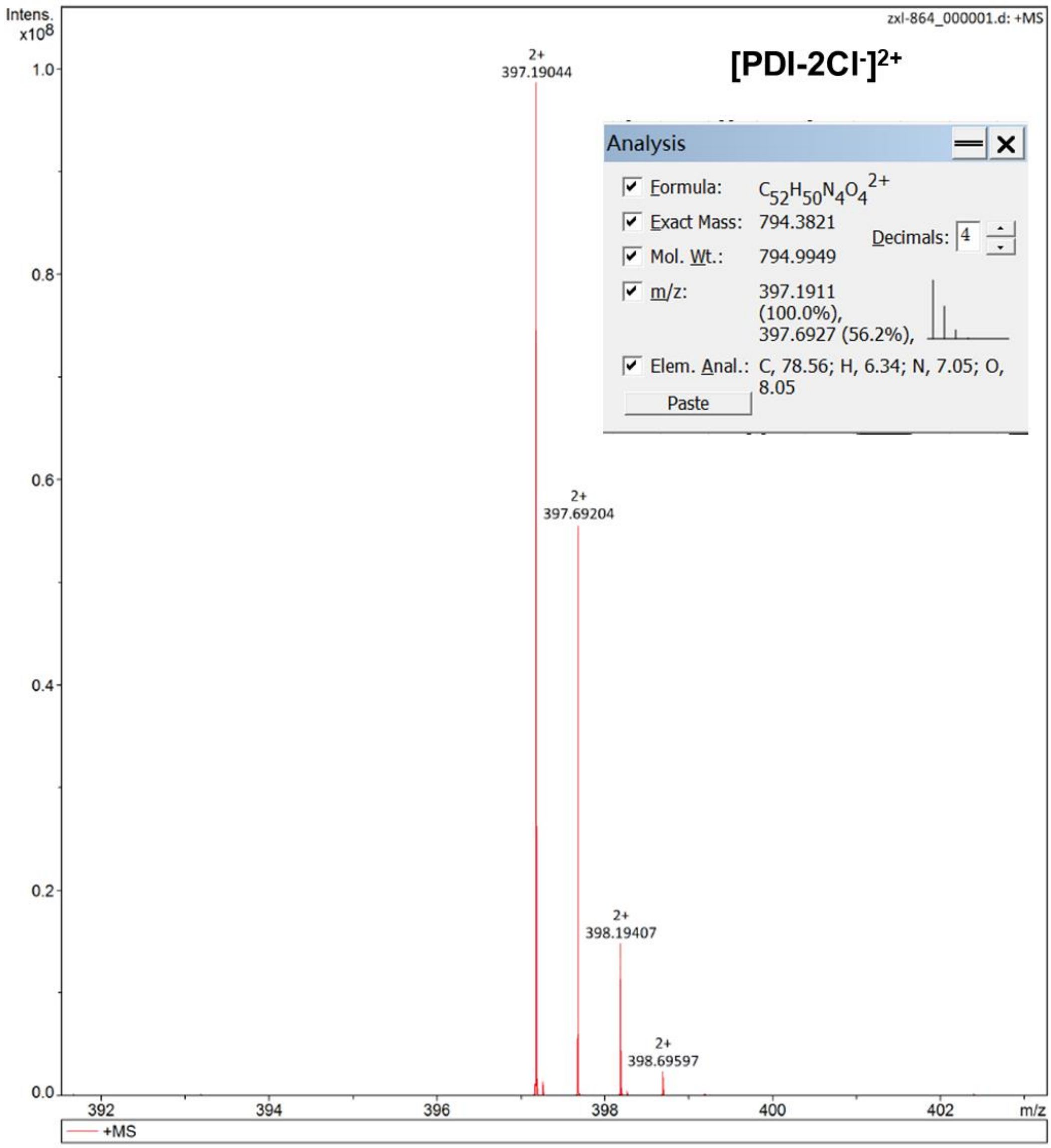

Figure S4: HRMS spectrum of PDI.

3.2 Characterization of PDI and PDI+2CB[7]

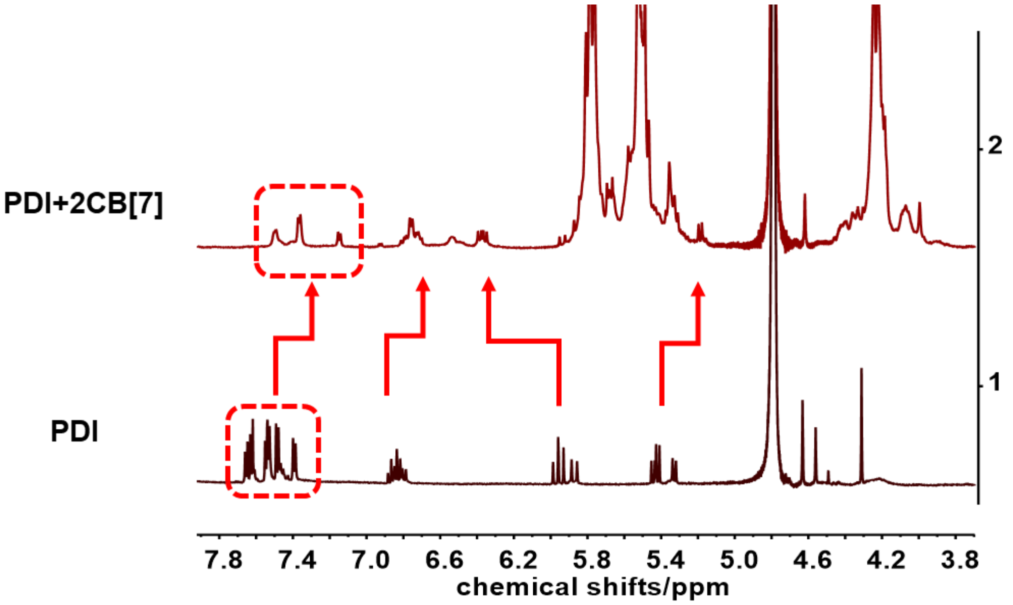

Figure S5: The change of ^{1}H NMR spectrum (600 MHz, 90% D_2O, 10% DMSO-d_6, 25 °C) of PDI and PDI+2CB[7].

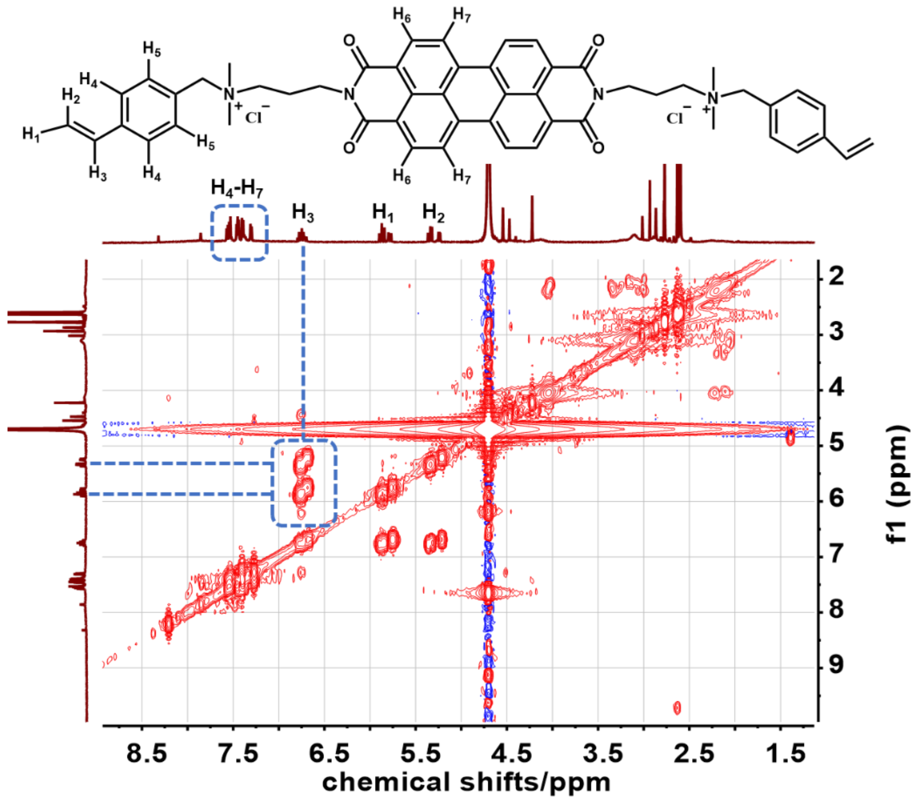

Figure S6: COSY spectra of PDI, 600 MHz, 90% D_2O, 10% DMSO-d_6, 25 °C.

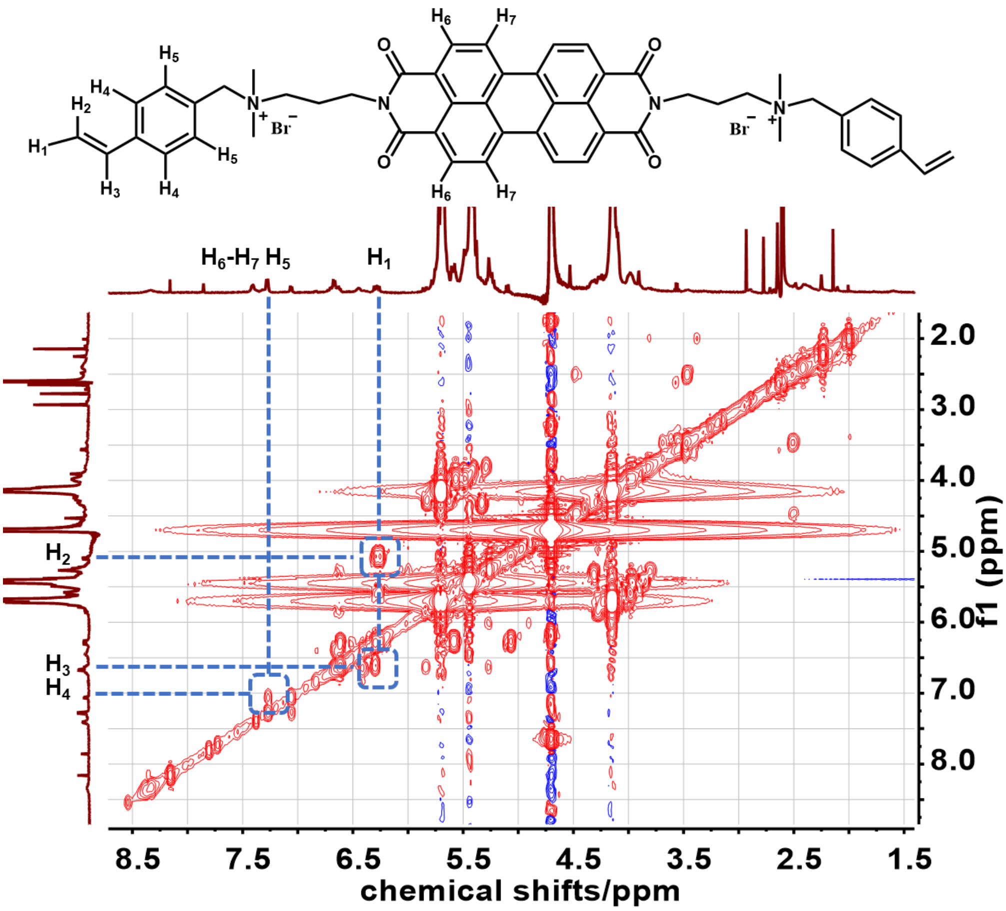

Figure S7: COSY spectra of PDI+2CB[7], 600 MHz, 90% D_2O, 10% DMSO-d_6, 25 °C.

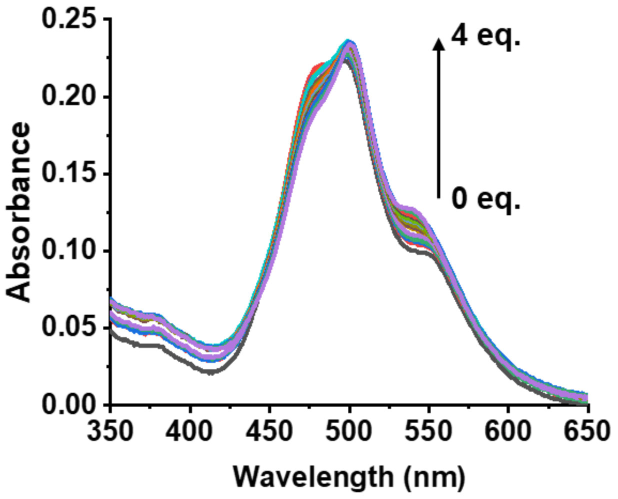

Figure S8: UV−vis spectral changes of PDI upon addition of CB[7] ([PDI] = 5.0 μM and [CB[7]] = 0-20.0 μM).

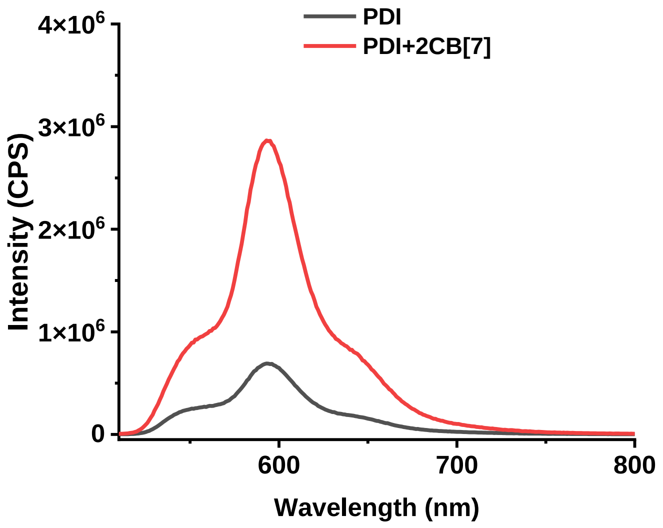

Figure S9: Fluorescence emission spectra of PDI and PDI+2CB[7]. [CB[7]] = 2[PDI] = 1.0 mM, [$Na_2S_2O_4$] = 2.0 mM.

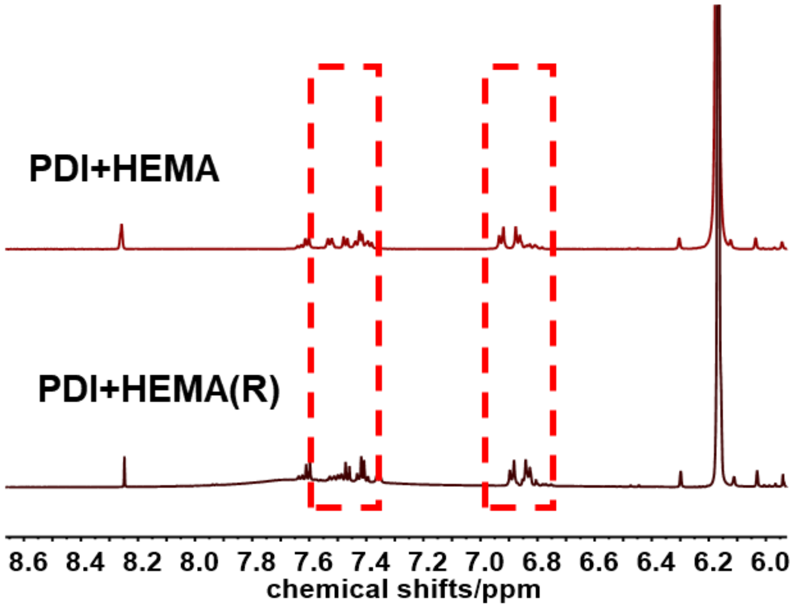

Figure S10: ^{1}H NMR spectrum (600 MHz, 90% D_2O, 10% DMSO-d_6, 25 ° C) of PDI+HEMA and PDI+HEMA(R). (R: reducing agent, $Na_2S_2O_4$)

3.3 The fluorescence intensity of 4T1 cells after different incubation times.

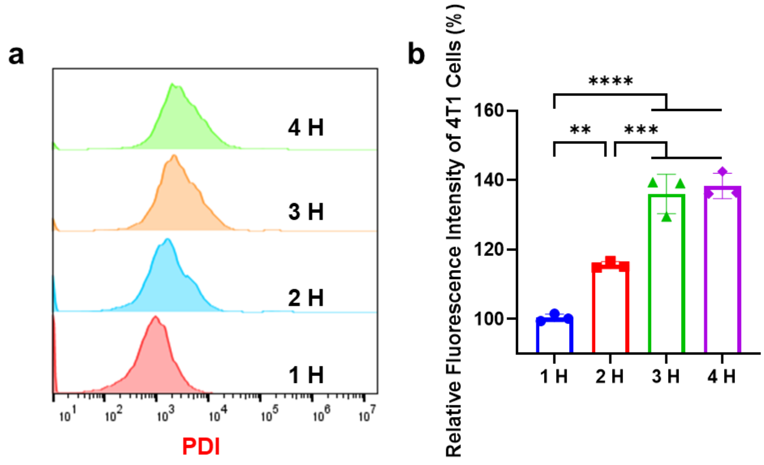

Figure S11: a Representative flow cytometry results of the fluorescence of 4T1 cells incubated with PDI+2CB[7]+HEMA for different time. **b** Quantitative result of the fluorescence intensity of PDI+2CB[7]+HEMA in 4T1 cells after different time of incubation analyzed by flow cytometry. [PDI] = [HEMA] = 12.5 μM, [CB[7]] = 25.0 μM.

3.4 Relative fluorescence intensity of 4T1 cells and HaCat cells after different treatments.

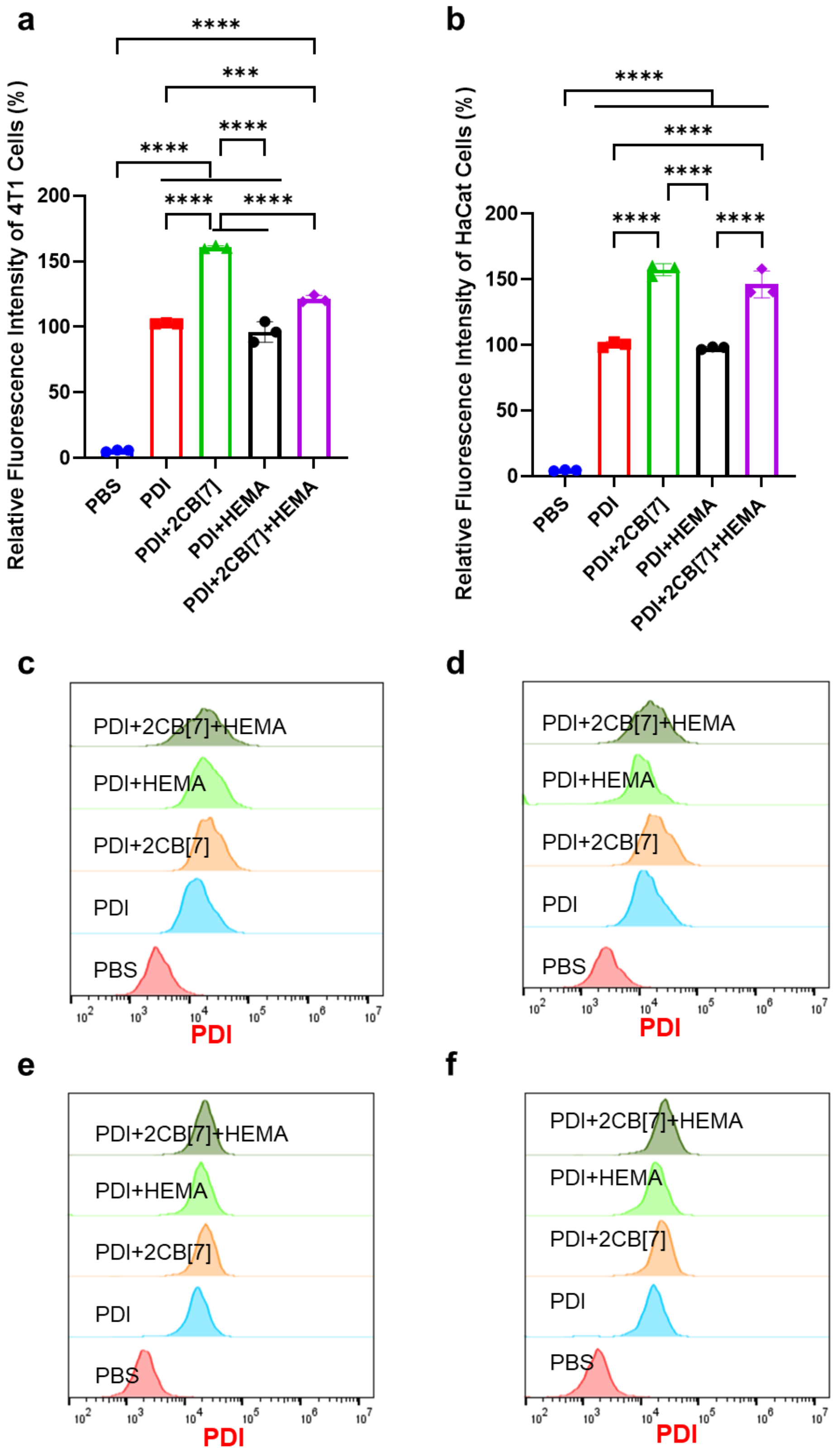

Figure S12: a Quantitative result of the fluorescence intensity of PDI+2CB[7]+HEMA in 4T1 cells analyzed by flow cytometry. [PDI] = [HEMA] = 12.5 μM, [CB[7]] = 25.0

μM. **b** Quantitative result of the fluorescence intensity of PDI+2CB[7]+HEMA in HaCat cells analyzed by flow cytometry. [PDI] = [HEMA] = 12.5 μM, [CB[7]] = 25.0 μM. **c, d** Flow cytometry results of 4T1 cells incubated with PBS, PDI, PDI+2CB[7], PDI+HEMA and PDI+2CB[7]+HEMA in hypoxic environment. [CB[7]] = 2[PDI] = 2[HEMA] = 25.0 μM. **e, f** Flow cytometry results of HaCat cells incubated with PBS, PDI, PDI+2CB[7], HEMA, PDI+HEMA and PDI+2CB[7]+HEMA in hypoxic environment. [CB[7]] = 2[PDI] = 2[HEMA] = 25.0 μM.

3.5 Bio-TEM images of HaCat cells after different treatments.

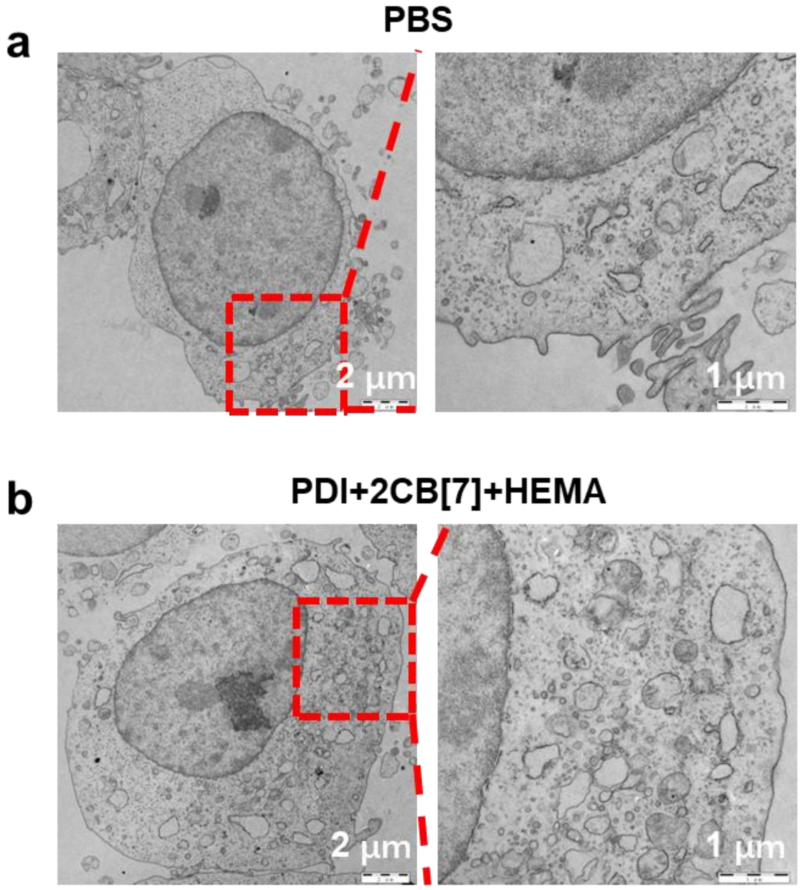

Figure S13: Biological transmission electron microscopy (Bio-TEM) images of HaCat cells incubated with **a** PBS and **b** PDI+2CB[7]+HEMA. [PDI] = [HEMA] = 12.5 μM, [CB[7]] = 25.0 μM.

3.6 Characterization of intracellular polymers.

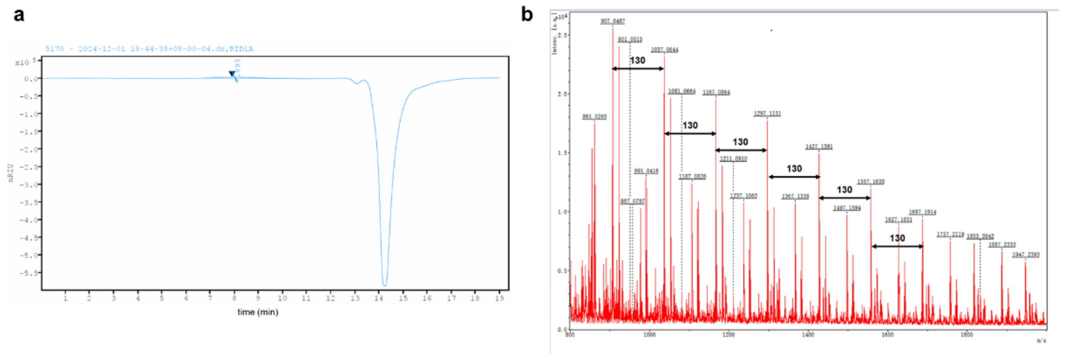

Figure S14: **a** GPC trace of 4T1 cell lysate. The experiments were repeated, independently for 3 times. **b** MALDI-TOF MS spectrum of intracellular polymer extracted from cell lysate.

3.7 Relative fluorescence intensity of 4T1 cells and HaCat cells after different incubation time.

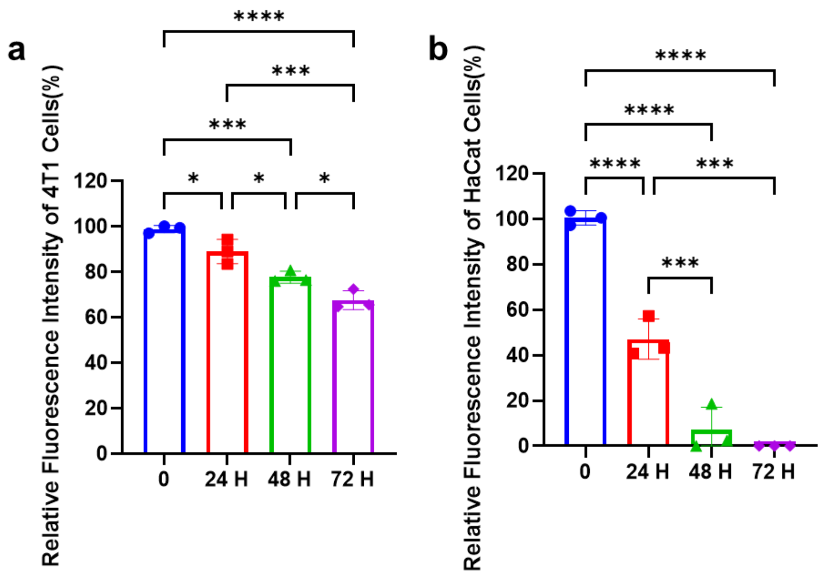

Figure S15: The analytical results of the fluorescence of **a** 4T1 cells and **b** HaCat cells incubated with PDI+2CB[7]+HEMA. 4T1 cells were incubated in hypoxic environment. [CB[7]] = 2[PDI] = 2[HEMA] = 25.0 μM.

3.8 Relative ROS levels in4T1 cells after different treatments.

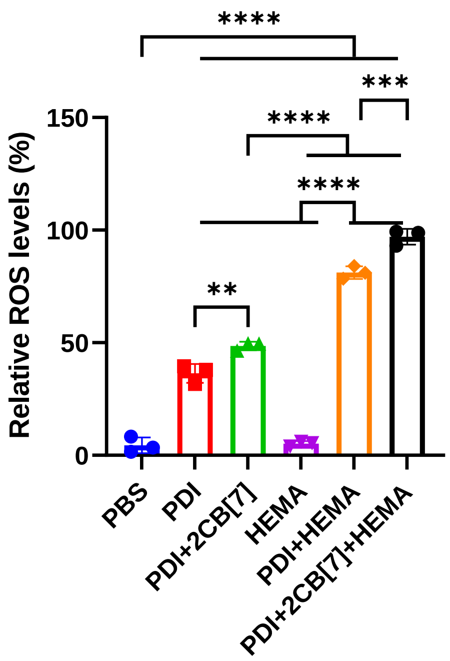

Figure S16: Analytical results of ROS generation in 4T1 cells with different treatments by flow cytometry. [CB[7]] = 2[PDI] = 2[HEMA] = 25.0 μM.

3.9 Relative ATP levels in4T1 cells after different treatments.

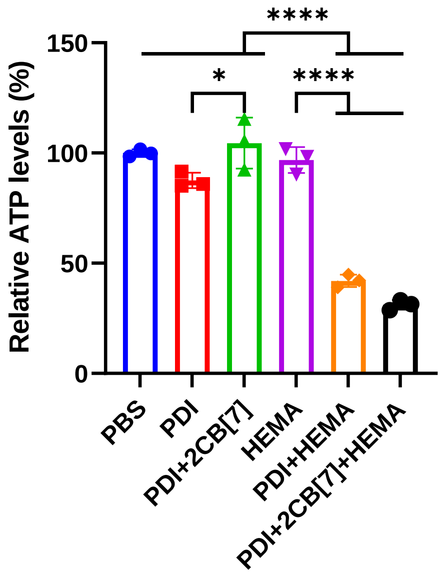

Figure S17: The relative ATP levels of 4T1 cells with different treatments. [CB[7]] = 2[PDI] = 2[HEMA] = 25.0 μM.

3.10 Relative Edu intensity in 4T1 cells after different treatments.

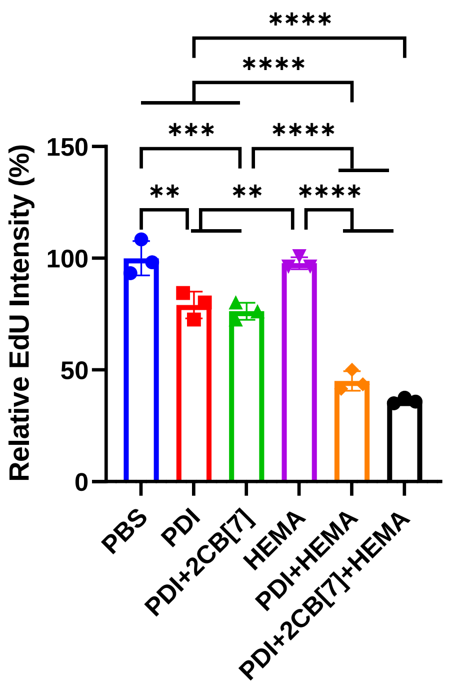

Figure S18: Analytical results of relative EdU intensity in 4T1 cells with different treatments by flow cytometry. [PDI] = [HEMA] = 12.5 μM, [CB[7]] = 25.0 μM.

3.11 The cell cycle of 4T1 cells after different treatments.

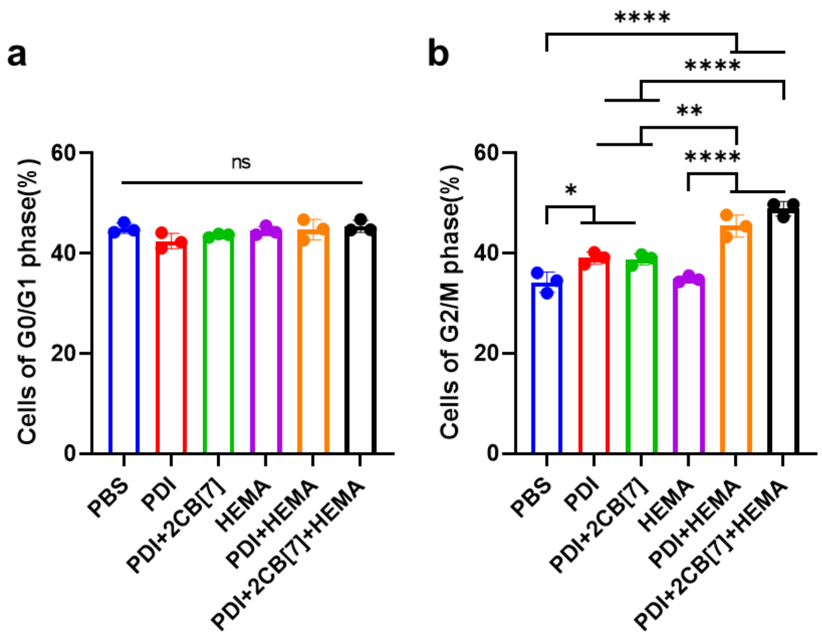

Figure S19: The cell cycle was investigated by treating the 4T1 cells with different treatments. [CB[7]] = 2[PDI] = 2[HEMA] = 25.0 μM. Treated cells were fixed with 70% ethanol overnight, treated with RNase A, stained by PI, and analyzed by flow cytometry.

3.12 The HIF-1α level of normal tissue.

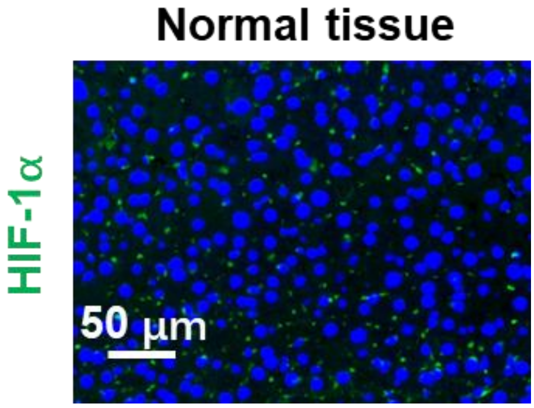

Figure S20: Fluorescence images of normal tissue from mice. Sections were stained with DAPI (blue), HIF-1α (green).

3.13 The safety evaluation.

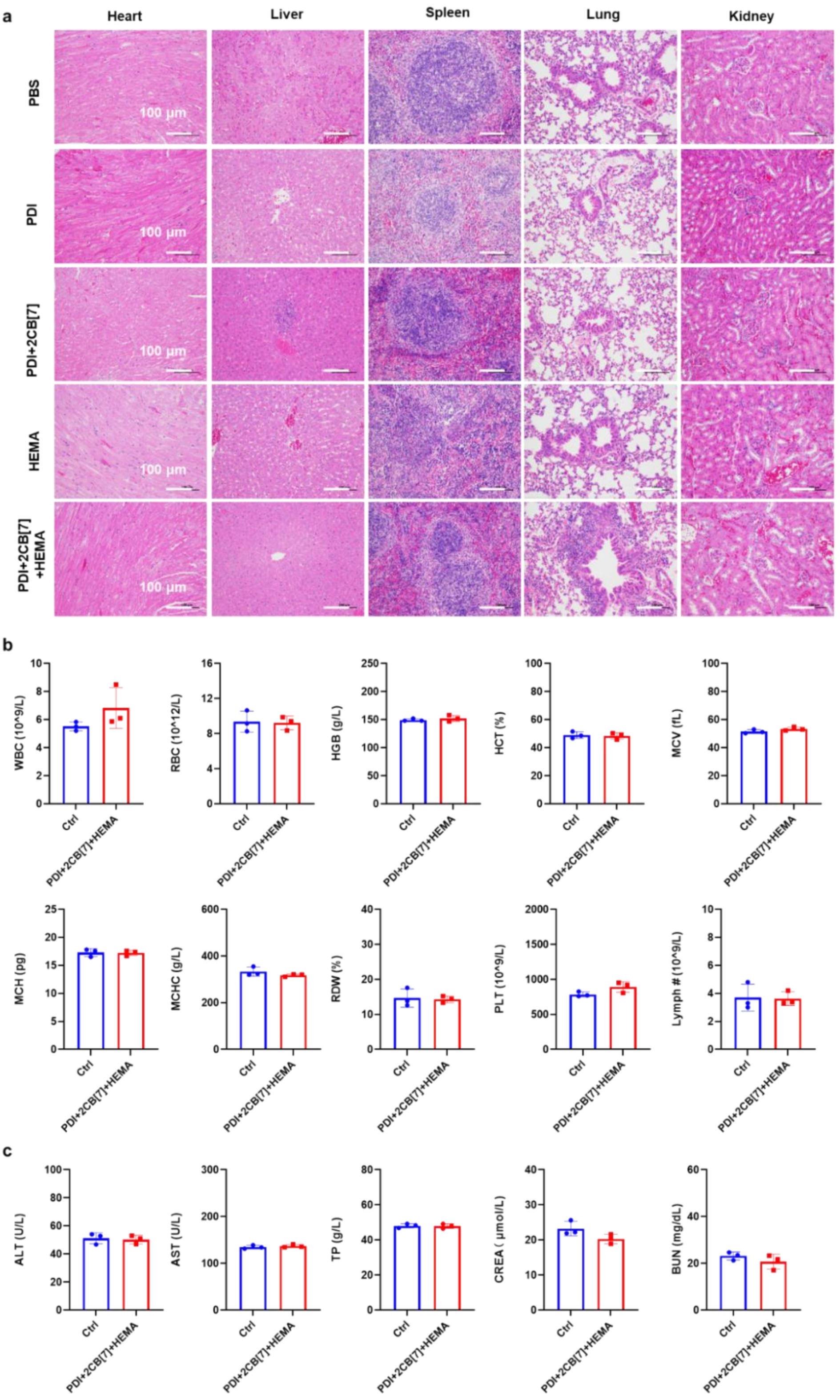

Supplementary Fig. 21: a 15 tumor-bearing mice were randomly divided into five groups: PBS, PDI, PDI+2CB[7], HEMA and PDI+2CB[7]+HEMA ([CB[7]] = 2[PDI] = 2[HEMA] = 25.0 μM, 100 μL). After 3 times of treatment, the organs (heart, liver,

spleen, lung and kidney) were collected for H&E staining. **b** Whole blood from healthy mice and PDI+2CB[7]+HEMA group were for studies of the routine blood tests. **c** Serum from healthy mice and PDI+2CB[7]+HEMA group were for studies of typical indicators of liver and kidney function.

4. References

(1) Yang, Y.; He, P.; Wang, Y.; Bai, H.; Wang, S.; Xu, J.-F.; Zhang, X. *Angew. Chem. Int. Ed.* **56**, 16239-16242 (2017).
